# Crystal structures and photophysical properties of mono- and dinuclear Zn^II^ complexes flanked by tri­ethyl­ammonium

**DOI:** 10.1107/S2056989024010302

**Published:** 2024-10-24

**Authors:** Hai Le Thi Hong, Hien Nguyen, Duong Trinh Hong, Ninh Nguyen Hoang, Khanh Nguyen Nhat, Luc Van Meervelt

**Affiliations:** a Department of Chemistry, Hanoi National University of Education, 136 Xuan Thuy, Cau Giay, Hanoi, Vietnam; b Institute of Natural Sciences, Hanoi National University of Education, 136 Xuan Thuy, Cau Giay, Hanoi, Vietnam; c Department of Chemistry, KU Leuven, Biomolecular Architecture, Celestijnenlaan 200F, Leuven (Heverlee), B-3001, Belgium; Universidad de la República, Uruguay

**Keywords:** crystal structure, Zn(II) complex, 8-hy­droxy­quinoline derivatives, Schiff base.

## Abstract

Two new zinc(II) complexes, tri­ethyl­ammonium di­chlorido­[2-(4-nitro­phen­yl)-4-phenyl­quinolin-8-olato]zinc(II), (C_6_H_16_N){Zn(C_21_H_13_N_2_O_3_)Cl_2_] (**ZnOQ**), and bis­(tri­ethyl­ammonium) {2,2′-[1,4-phenyl­enebis(nitrilo­methyl­idyne)]diphenolato}bis­[di­chlorido­zinc(II)], (C_6_H_16_N)_2_[Zn_2_(C_20_H_14_N_2_O_2_)Cl_4_] (**ZnBS**), were synthesized and their structures were determined using ESI–MS spectrometry, ^1^H NMR spectroscopy, and single-crystal X-ray diffraction. The results showed that the ligands 2-(4-nitro­phen­yl)-4-phenyl­quinolin-8-ol (**HOQ**) and *N*,*N*′-bis­(2-hy­droxy­benzyl­idene)benzene-1,4-di­amine (**H_2_BS**) were deprotonated by tri­ethyl-amine, forming the counter-ion Et_3_NH^+^, which inter­acts *via* an N—H⋯O hydrogen bond with the ligand. The Zn^II^ atoms have a distorted trigonal–pyramidal (**ZnOQ**) and distorted tetra­hedral (**ZnBS**) geometries with a coord­ination number of four, coordinating with the ligands *via* N and O atoms. The N atoms coordinating with Zn^II^ correspond to the heterocyclic nitro­gen for the **HOQ** ligand, while for the **H_2_BS** ligand, it is the nitro­gen of the imine (CH=N). The crystal packing of **ZnOQ** is characterized by C—H⋯π inter­actions, while that of **ZnBS** by C—H⋯Cl inter­actions. The emission spectra showed that **ZnBS** complex exhibits green fluorescence in the solid state with a small band-gap energy, and the **ZnOQ** complex does exhibit non-fluorescence.

## Abstract 2

Two new zinc(II) complexes, C_21_H_13_Cl_2_N_2_O_3_Zn·C_6_H_16_N (**ZnOQ**) and C_20_H_14_Cl_4_N_2_O_2_Zn·2C_6_H_16_N (**ZnBS**), were synthesized and their structures were determined using ESI–MS spectrometry, ^1^H NMR spectroscopy, and single-crystal X-ray diffraction.

## 1. Chemical context

Numerous Zn^II^ complexes have attracted inter­est from many scientists and have been used in various applications, such as biological sensors (Liu *et al.*, 2020 ▸; He *et al.*, 2020 ▸), anti­microbial agents (Kargar *et al.*, 2021*a* ▸*b* ▸), anti­cancer drugs (Du *et al.*, 2023 ▸) and particularly in luminescent materials for organic light-emitting diode (OLED) devices (Gusev *et al.*, 2019 ▸, 2021 ▸; Rashamuse *et al.*, 2023 ▸). Zn^II^ complexes are noted for their impressive fluorescence and cost-effectiveness in OLED applications. Among all ligands, 8-hy­droxy­quinoline is a classical one. It has the ability to form a five-membered ring with the metal center *via* N and O atoms, which appeals to many scientists from all over the world (Côrte-Real *et al.*, 2023 ▸; Harmošová *et al.*, 2023 ▸). In order to improve the photophysical properties of Zn^II^ complexes with 8-hy­droxy­quinoline, many strategies have been conducted to synthesize new neutral Zn^II^ complexes including different substituents at various positions on 8-hy­droxy­quinoline (Singh *et al.*, 2018 ▸), with the approach of extending the π-conjugation system with aryl substituents to increase photoluminiscence quantum yield (PLQY) and to shift the emission to blue yielding potential results (Harmošová *et al.*, 2023 ▸; Jianbo *et al.*, 2018 ▸; Hien *et al.*, 2024 ▸). In particular, a series of six new Zn^II^ complexes bearing diaryl-8-hy­droxy­quinoline were synthesized, indicating that electron-donating groups (like OCH_3_) enhance the PLQY, while electron-withdrawing groups (like NO_2_) show the opposite result (Hien *et al.*, 2024 ▸). These complexes are synthesized by direct reaction between ZnCl_2_ and the ligands to obtain neutral complexes [Zn(OQ)_2_], in which Zn^II^ coordinates with deprotonated 8-hy­droxy­quinoline *via* N and O atoms, as in previous publications. However, in this work, upon the reaction of 2-(4-nitro­phen­yl)-4-phenyl­quinolin-8-ol (**HOQ**) with ZnCl_2_ in the presence of tri­ethyl­amine, an ionic complex with the mol­ecular formula [Et_3_NH][Zn(OQ)Cl_2_] (**ZnOQ**) was obtained, in which the ratio of Zn^II^ and ligand is 1:1 instead of 1:2 as in the published Zn^II^ complexes (Singh *et al.*, 2018 ▸; Hien *et al.*, 2024 ▸). Furthermore, the same reaction condition between ZnCl_2_ and a similar NO-Schiff base ligand, namely *N*,*N*′-bis­(2-hy­droxy­benzyl­idine)benzene-1,4-di­amine (**H_2_BS**) gives a similar ion complex [Zn_2_(BS)Cl_2_][Et_3_NH]_2_ (**ZnBS**).
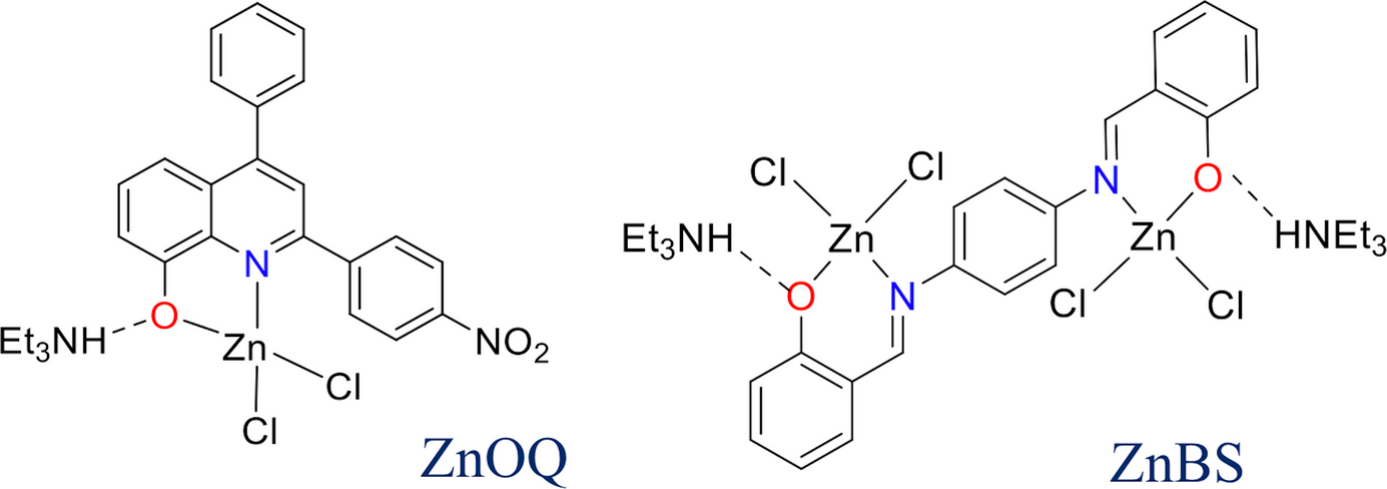


In this report, the ligands **HOQ** and **H_2_BS** were successfully prepared, and characterized. Furthermore, two complexes **ZnOQ** and **ZnBS** were also successfully prepared, isolated and characterized by ESI–MS and ^1^H NMR, and the crystal structures of the complexes were elucidated. The optical properties of the ligands and complexes were studied using absorption and emission spectra in both solid state and in solution in di­methyl­sulfoxide (DMSO) or tetra­hydro­furan (THF) solvents.

## 2. Structural commentary

**Figure 1:**
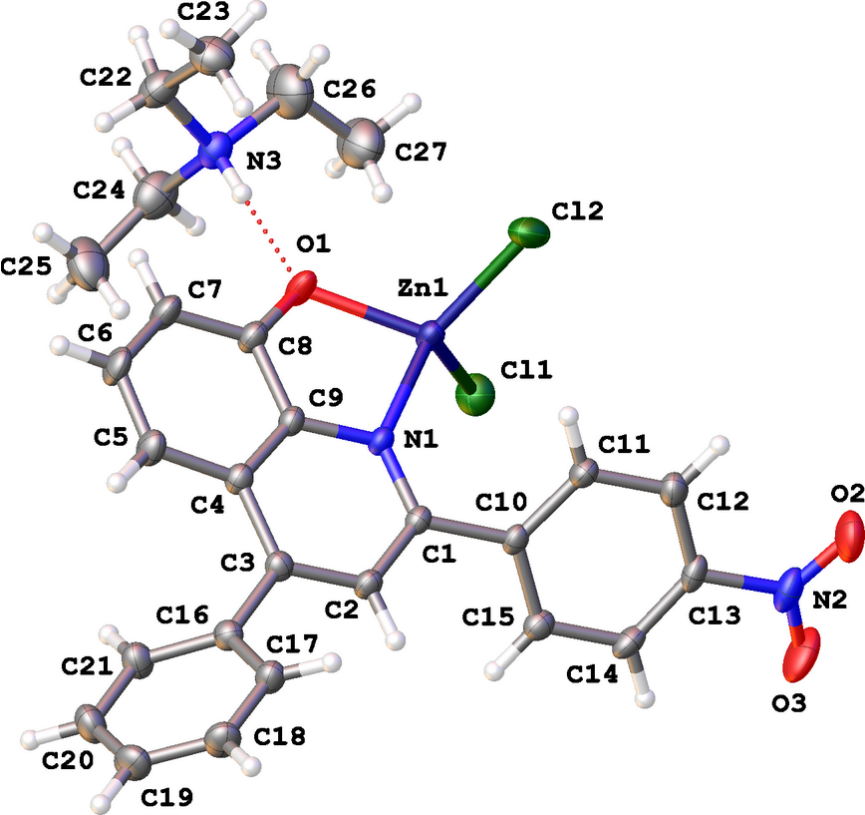
The mol­ecular structure of **ZnOQ** showing the atom-labeling scheme and displacement ellipsoids at the 30% probability level. The N—H⋯O hydrogen bond is shown as a red dashed line.

The mononuclear complex **ZnOQ** crystallizes in the monoclinic space group *P*2_1_/*c* with one mol­ecule in the asymmetric unit (Fig. 1 ▸). The Zn^II^ atom coordinates to the N and O atoms of a deprotonated 8-hydoxyquinoline derivative and two chlorine atoms with a distorted trigonal–pyramidal geometry (τ_4_ parameter is 0.86; Yang *et al.*, 2007 ▸). The negative charge of the complex is compensated by the inter­action with tri­ethyl­ammonium *via* an N—H⋯O hydrogen bond (Table 1 ▸). The Zn atom is part of a five-membered ring and is located 0.081 (1) Å above the planar quinoline plane (r.m.s. deviation = 0.058 Å), which makes dihedral angles of 50.88 (12) and 46.95 (13)° with the C10–C15 and C16-C21 phenyl rings, respectively. The mutual angle between the two phenyl rings is 79.42 (16)°. The plane of the nitro group makes an angle of 14.38 (19)° with the C10–C15 phenyl ring.

**Figure 2:**
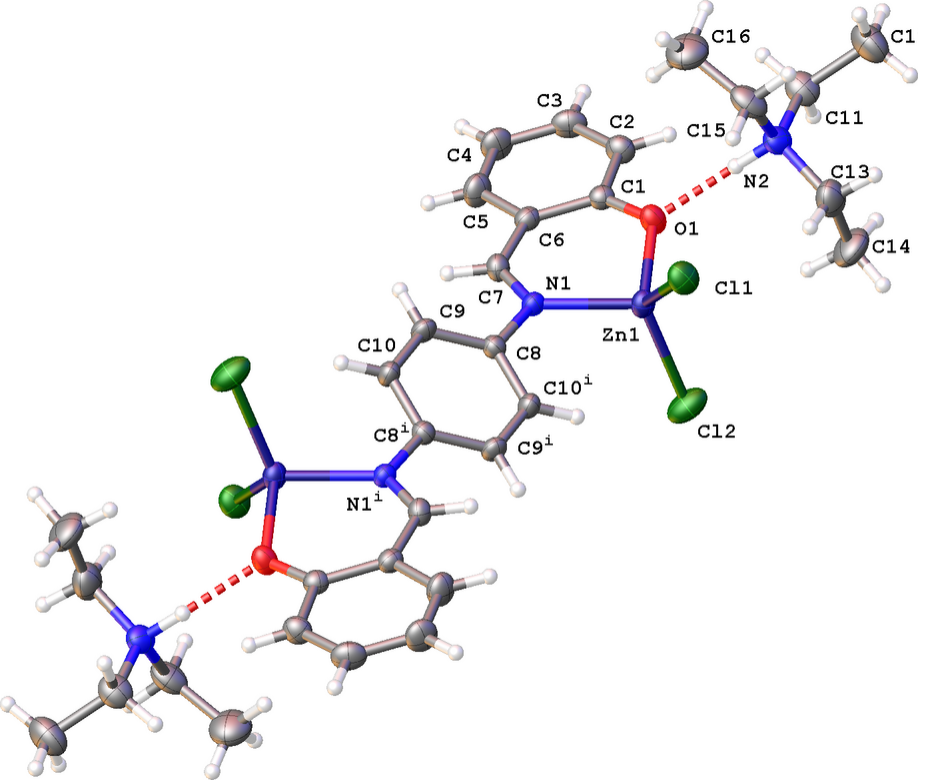
The mol­ecular structure of **ZnBS** showing the atom-labeling scheme and displacement ellipsoids at the 30% probability level. The N—H⋯O hydrogen bonds are shown as a red dashed line. Symmetry code: (i) −*x* + 1, −*y* + 1, −*z* + 2.

The dinuclear complex **ZnBS** also crystallizes in the monoclinic space group *P*2_1_/*c* but with half a mol­ecule in the asymmetric unit (Fig. 2 ▸). The second half is generated by inversion symmetry. The complex is flanked at both ends by a tri­ethyl­ammonium moiety *via* an N—H⋯O inter­action (Table 2 ▸). The Zn^II^ coordination sphere resembles that observed in **ZnOQ**, but is now inter­mediate between trigonal–pyramidal and tetra­gonal geometries (τ_4_ parameter is 0.91). The Zn^II^ atom is part of a six-membered ring and is located 0.405 (3) Å above the best plane through atoms C1–C7/O1/N1 (r.m.s. deviation = 0.027 Å). The inter­planar angle between the aromatic rings is 33.4 (2)°. The stereochemistry of the C7=N1 bond is *E*, as illustrated by the torsion angle C6—C7=N1—C8 of 176.9 (4)°.

## 3. Supra­molecular features

**Figure 3:**
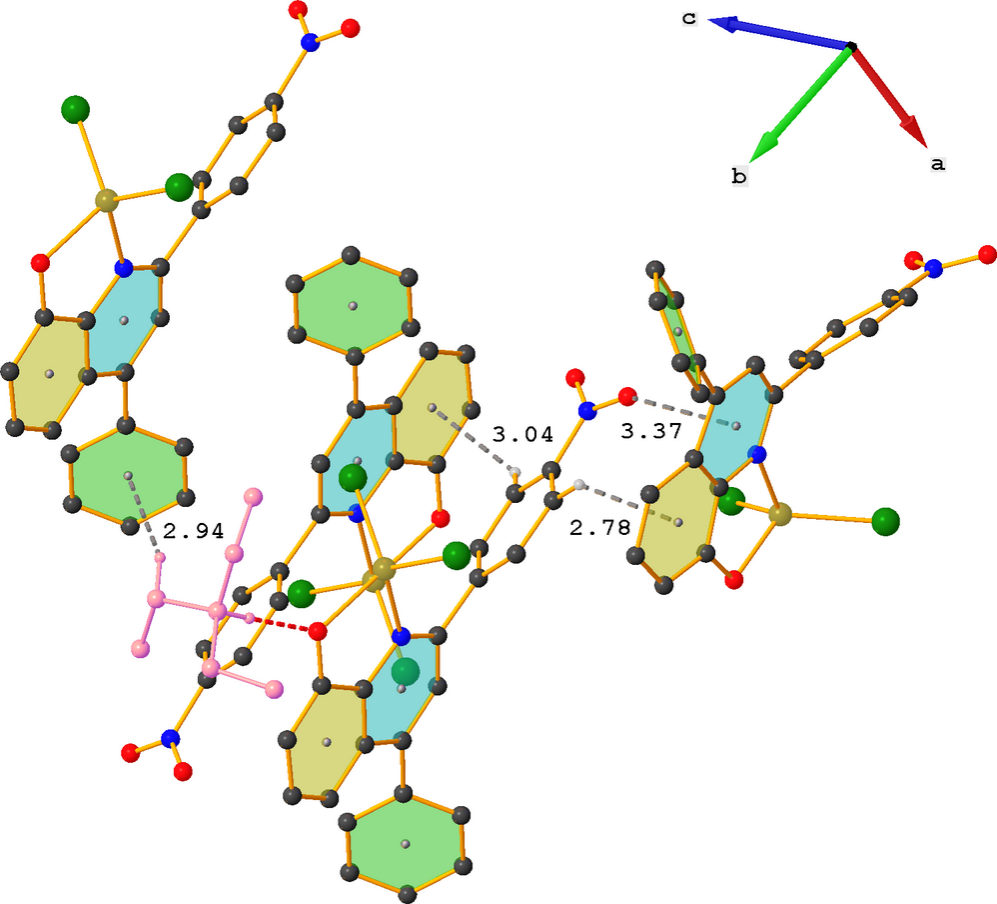
Partial crystal packing of **ZnOQ** showing the C—H⋯π and N—O⋯π inter­actions as gray dashed lines. The N—H⋯O hydrogen bond is shown as a red dashed line. Further details are given in Table 1 ▸. For clarity, hydrogen atoms not involved in hydrogen bonding are omitted and the tri­ethyl­ammonium ion is shown in pink.

Despite the presence of aromatic rings in **ZnOQ**, no π–π stacking is observed in the crystal packing. However, the phenyl part of the quinoline ring system (C4–C9) and one of the phenyl rings (C16–C21) participate in three C—H⋯π inter­actions (Table 1 ▸, Fig. 3 ▸). Centrosymmetric dimers are formed by inter­action of C14—H14 with a nearby C4–C9 ring. In addition, the other side of the nitro­phenyl ring (C12—H12) also inter­acts with a close by C4–C9 ring. The last inter­action involves the tri­ethyl­ammonium ion, with C24—H24*A* inter­acting with a neighboring C16–C21 ring, resulting in chain formation along the *a*-axis direction. One of the nitro oxygen atoms (O2) shows an O⋯π inter­action with the pyridine part of the quinoline ring system [O2⋯*Cg*2^i^ = 3.372 (3) Å; *Cg*2 is the centroid of the N1/C1–C4/C9 ring; symmetry code: (i) −*x* + 1, *y* − 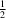, −*z* + 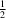].

**Figure 4:**
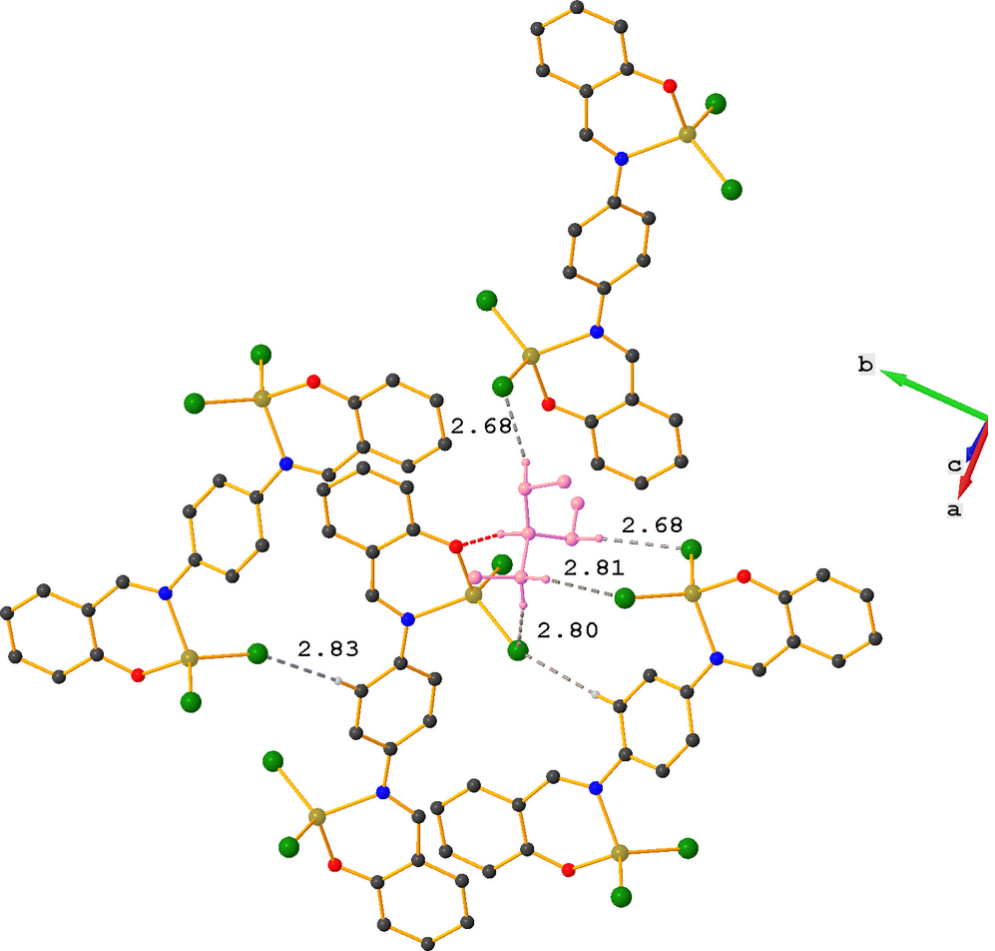
Partial crystal packing of **ZnBS** showing the C—H⋯Cl inter­actions as gray dashed lines. The N—H⋯O hydrogen bond is shown as a red dashed line. Further details are given in Table 2 ▸. For clarity, hydrogen atoms not involved in hydrogen bonding are omitted and the tri­ethyl­ammonium ion is shown in pink.

**Figure 5:**
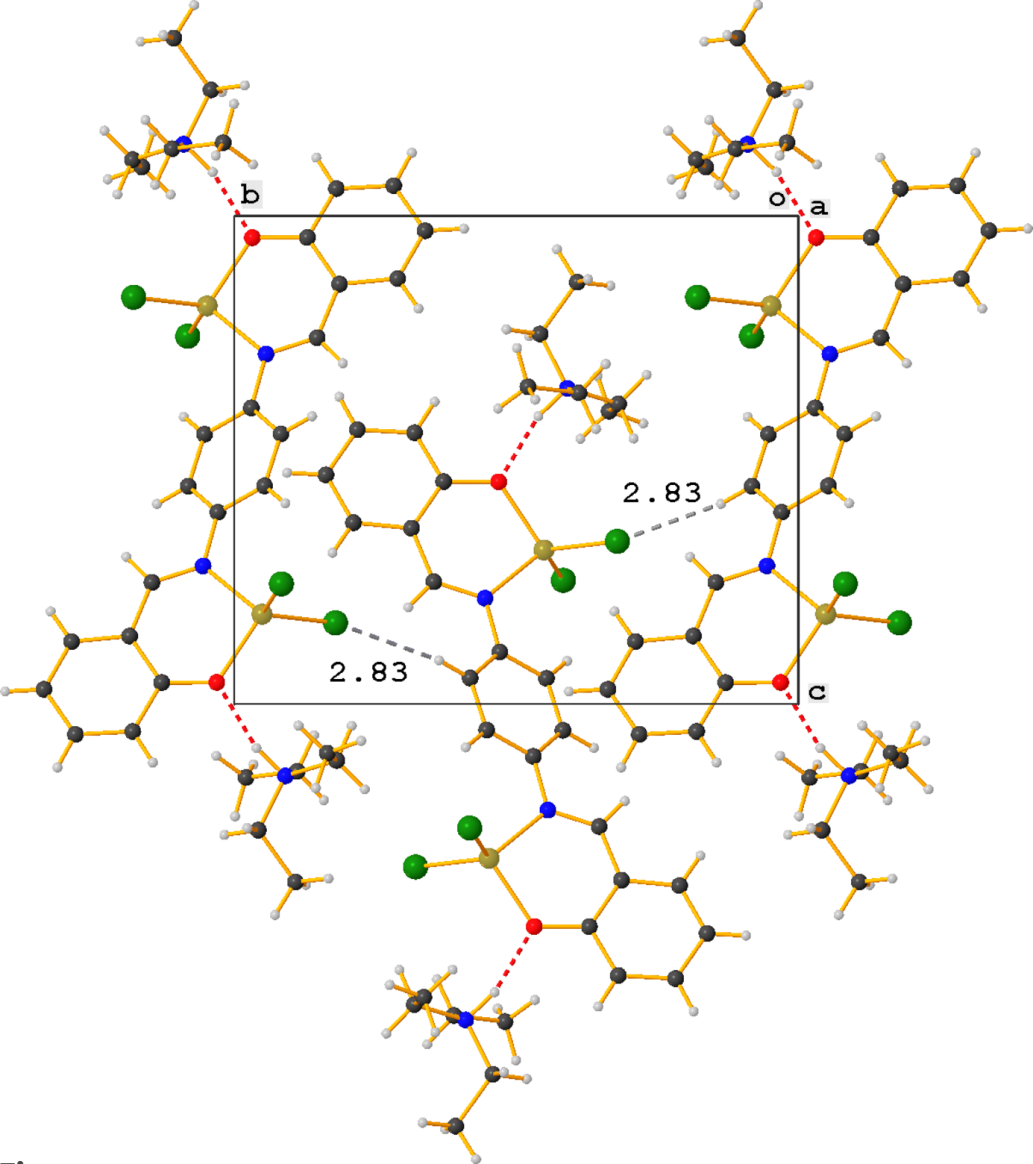
Chain formation in the *b*-axis direction by C—H⋯Cl inter­actions (gray dashed lines) in the crystal packing of **ZnBS**.

In contrast to **ZnOQ**, the crystal packing in **ZnBS** is characterized by C—H⋯Cl inter­actions (Table 2 ▸, Fig. 4 ▸). The tri­ethyl­ammonium ion plays an important role in these inter­actions and acts as a stabilizing glue between three complexes *via* one N—H⋯O and four C—H⋯Cl inter­actions. The fifth C—H⋯Cl inter­action is between an H atom of the central phenyl ring (H9) and a nearby chlorine atom (Cl1), which results in the formation of chains running in the *b*-axis direction (Fig. 5 ▸).

## 4. Database survey

**Figure 6:**
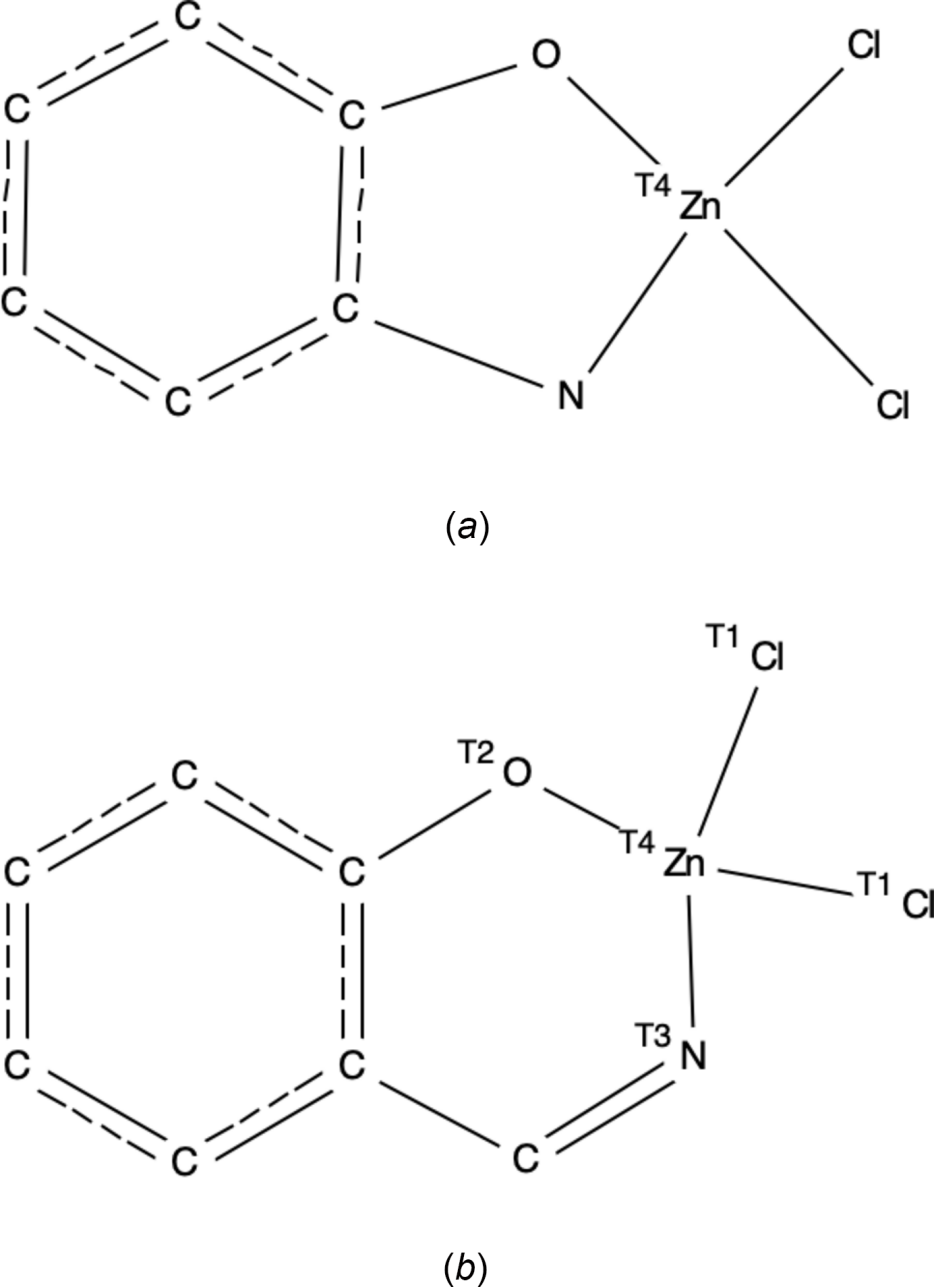
Search fragments used in Conquest to perform the CSD survey: (*a*) five-membered ring fragment present in **ZnOQ**, (*b*) six-membered ring fragment present in **ZnBS**.

A search in the Cambridge Structural Database (CSD, Version 5.45, last update September 2024; Groom *et al.*, 2016 ▸) for the five-membered ring fragment shown in Fig. 6 ▸*a* (comparable to a part of **ZnOQ**) resulted in three hits, CSD refcodes MOXFOX, MOXPEX and MOXPIB (Samanta *et al.*, 2019 ▸). In these structures, the deviation of the Zn^II^ atom from the best plane through the C, N and O atoms of the fragment (ranging between 0.145 and 0.195 Å) is comparable to that observed for **ZnOQ** [0.203 (3) Å]. The negative charge of the complexes is compensated by a second protonated ligand.

A similar search for the six-membered ring fragment shown in Fig. 6 ▸*b* (comparable to a part of **ZnBS**) resulted in 63 hits. The deviation of the Zn^II^ atom from the best plane through the C, N and O atoms of the fragment shows a large variation between 0.003 and 0.927 Å [mean value is 0.327 Å, 0.405 (3) Å for **ZnBS**].

Of the 1876 crystal structures containing a tri­ethyl­ammonium ion in the CSD, the N—H group inter­acts with an O atom in 383 structures (133 organic and 250 coordination compounds).

A quick search for a nitro group inter­action with a phenyl groups gives 6252 hits for an O⋯*Cg* distance shorter than 3.5 Å (*Cg* is the centroid of the phenyl ring).

## 5. Photophysical properties

**Figure 7:**
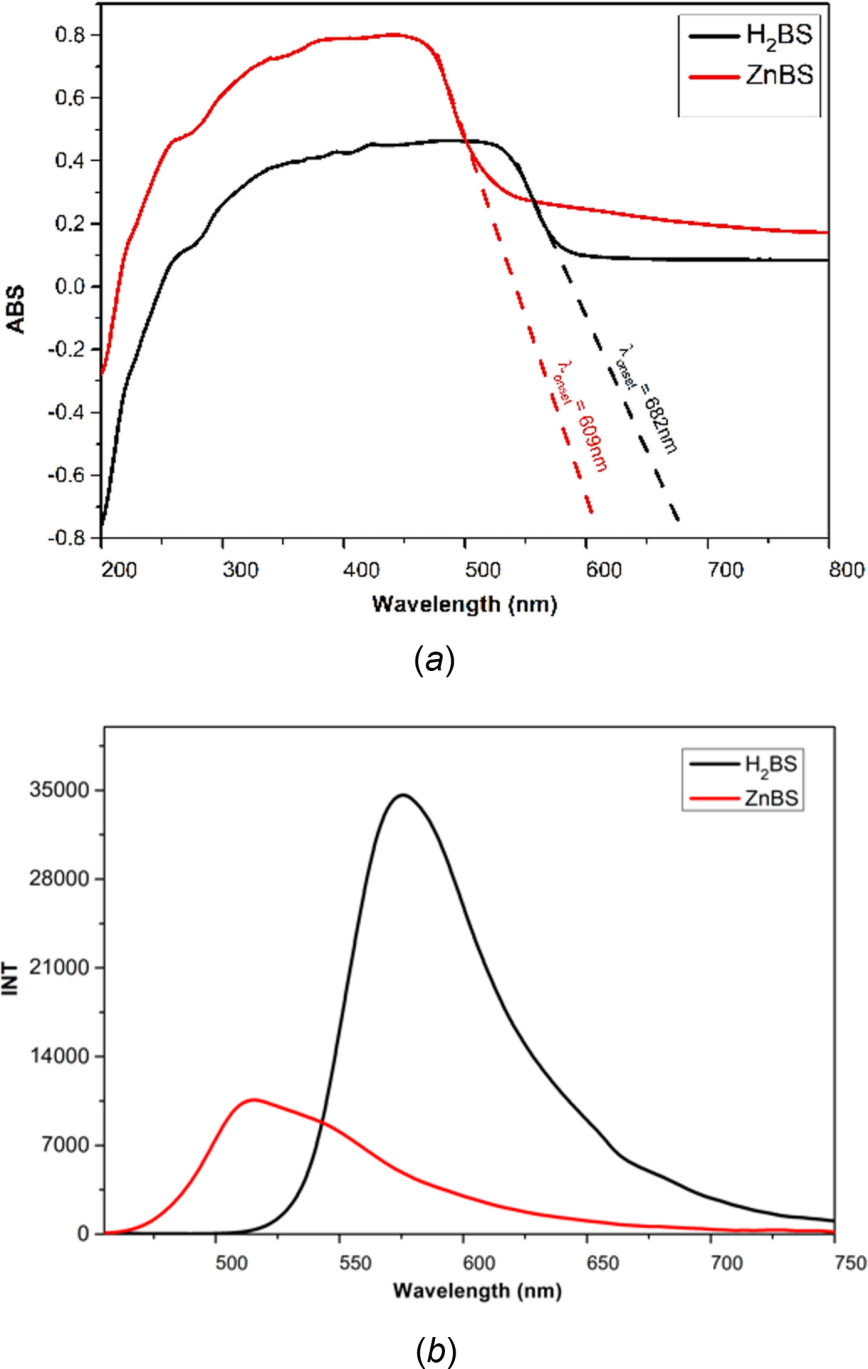
(*a*) Absorption and (*b*) emission spectra in the solid state at λ_ex_ = 425 nm of **H_2_BS** and **ZnBS**.

The absorption and emission spectra at room temperature of both ligands and complexes in DMSO or THF solvents at a concentration of 10 µ*M* (**H_2_BS**, **ZnBS**)**;** 50 µ*M* (**HOQ; ZnOQ**) and in the solid state (**H_2_BS**, **ZnBS)** are listed in Table 3 ▸. In the absorption spectra, **H_2_BS** and **ZnBS** (Fig. S9) show an absorption band at 371–372nm in both solvents, while two absorption bands were observed at 243–265nm and 295–312nm for **HOQ** and **ZnOQ** (Fig. S4), corresponding to π→π* or n→π* transitions. The results of the solid-state electron absorption spectrum of **H_2_BS** and **ZnBS** (Fig. 7 ▸*a*) show that the band-gap energies of **H_2_BS** and **ZnBS**, calculated according to the equation *E*_gap_ = *hc*/λ_onset_ (UV–vis) (Chiyindiko *et al.*, 2022 ▸) are approximately 1.8 eV and 2.0 eV, respectively, which has potential for applications in OLED devices (Dumur, 2014 ▸; Lakshmanan *et al.*, 2018 ▸).

The emission spectra of the examined complexes in DMSO and THF solvents demonstrate that all compounds show no fluorescence (Figs. S5 and S10). However, in the solid state, **H_2_BS** fluorescences at 575 nm with an intensity of approximately 35000 a.u., while the emission wavelength of **ZnBS** is 515 nm with an intensity of about 10000 a.u., showing a blue shift compared to the ligand with Δλ = 60 nm (Fig. 7 ▸*b* and S11).

## 6. Synthesis and crystallization

**Figure 8:**
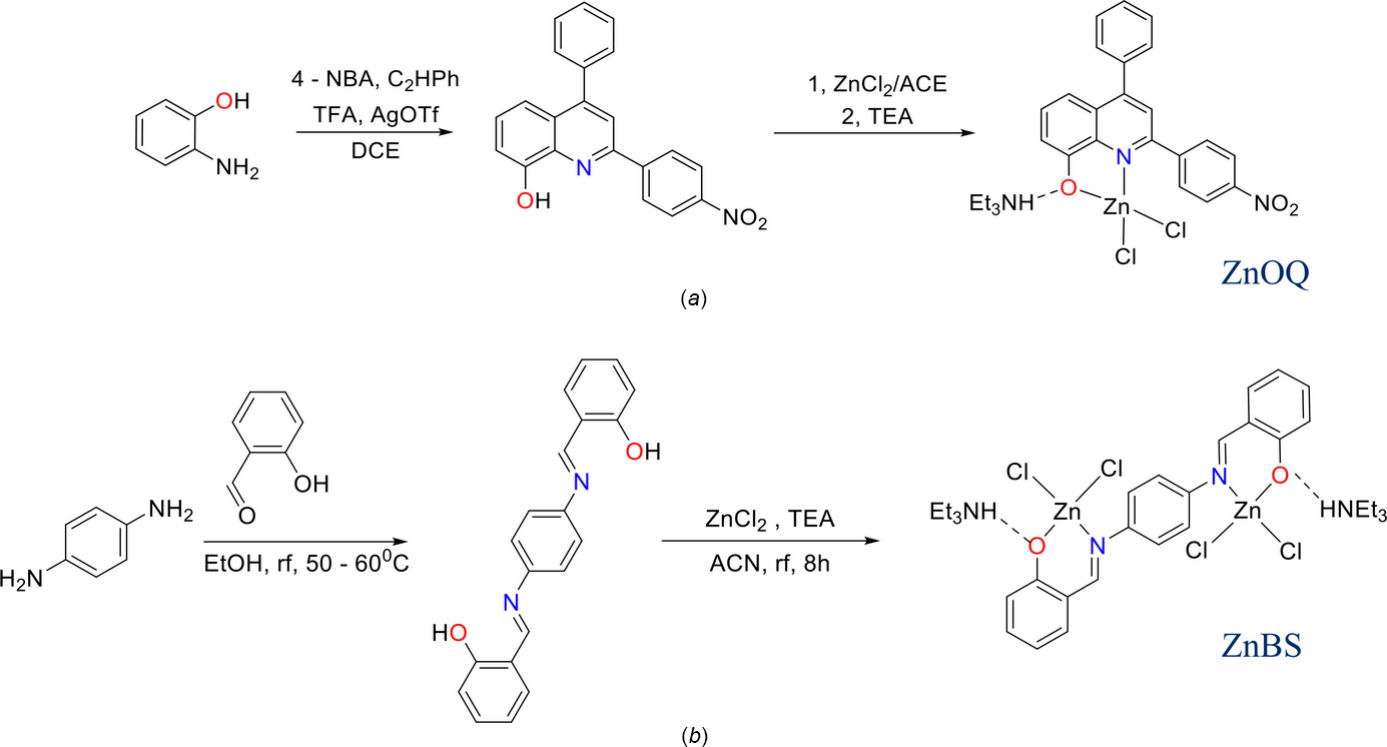
Synthesis of the complexes (*a*) **ZnOQ** and (*b*) **ZnBS**.

The reaction sequence for **ZnOQ** and **ZnBS** is shown in Fig. 8 ▸. The ligands **HOQ** and **H_2_BS** were synthesized according to modified procedures described by Yu *et al.* (2018 ▸; for **HOQ**) and Das & Ghosh (1998 ▸; for **H_2_BS**).

**Synthesis of HOQ**

A mixture of *ortho*-amino­phenol (120 mg, 1.1 mmol), 4-nitro­benzaldehyde (151 mg, 1 mmol), phenyl­acetyl­ene (139 mg, 1.2 mmol), AgOTf (13 mg, 0.5 mol%) and TFA (456 mg, 400 mol%) in 4 mL of di­chloro­ethane was heated to 353 K for 24 h. After cooling, the reaction mixture was diluted with 15 mL of ethyl acetate and extracted three times with 10 mL of saturated NaHCO_3_ solution. Then, it was dried over anhydrous sodium sulfate, filtered, and concentrated *in vacuo*. The resulting residue was purified by silica gel chromatography using hexa­ne/ethyl acetate (*v*/*v* = 19:1) as eluent. **HOQ** was obtained as a yellow solid with a yield of 52%.

^1^H NMR (600 MHz, chloro­form-*d_1_*, δ ppm): 8.41 (*br*, 1H, OH), 8.39 [*d*, ^3^*J*(H,H) = 9.0 Hz, 2H, Ar-H], 8.36 [*d*, ^3^*J*(H,H) = 9.0 Hz, 2H, Ar-H], 7.90 (*s*, 1H, Ar-H), 7.57 [*d*, ^3^*J*(H,H) = 4.5 Hz, 4H, Ar-H], 7.56–7.54 (*m*, 1H, Ar-H), 7.48–7.43 (*m*, 2H, Ar-H), 7.26 (*ov*, 1H, Ar-H).

The ^1^H NMR spectrum of **HOQ** is given in Fig. S1.

**Synthesis of [(Et_3_NH)ZnCl_2_(OQ)] (ZnOQ)**

A reaction mixture consisting of ligand **HOQ** (32 mg, 0.1 mmol), zinc(II) chloride (55 mg, 0.1 mmol) and 5 mL of acetone was stirred at room temperature and 25 µL of tri­ethyl­amine were added to the reaction vessel and stirred for 6 h to obtain an orange solution. Evaporation of the solution gave orange crystals (yield 67%).

^1^H NMR (600 MHz, *d*_6_–DMSO, δ ppm): 8.44 (*m*, 4H, Ar-H), 7.64 (*s*, 1H, Ar-H), 7.55 (*m*, 6H, Ar-H), 7.43 (*m*, 1H, Ar-H), 7.15 (*d*, 1H, Ar-H), 6.85 (*m*, 1H, NH), 3.31 (*q*, 6H, CH_2_), 1,40 (*t*, 9H, CH_3_). ESI–MS: 787.5 (100%, Zn(OQ)_2_ + ACN + H^+^).

The ^1^H NMR and ESI–MS spectra of **ZnOQ** are given in Figs. S2 and S3, respectively.

**Synthesis of H_2_BS**

A mixture of *p*-phenyl­enedi­amine (108 mg, 1 mmol) and salicyl­aldehyde (122 mg, 1 mmol) in 10 mL of ethanol was heated to 333 K for 5 h to obtain the red–orange solid **H_2_BS**, which was washed with hot ethanol, with a yield of 85%.

^1^H NMR (600 MHz, DMSO-*d_6_*, δ ppm): 13.00 (*s*, 1H, OH), 9.00 (*s*, 1H, CH_imine_), 7.68 (*dd*, 1H, Ar-H), 7.54 (*s*, 2H, Ar-H), 7.43 (*m*, 1H, Ar-H), 6.95 (*m*, 2H, Ar-H).

The ^1^H NMR spectrum of **H_2_BS** is given in Fig. S6.

**Synthesis of [(Et_3_NH)_2_Zn_2_Cl_4_(BS)] (ZnBS)**

A reaction mixture consisting of ligand **H­_2_BS** (32 mg, 0.1 mmol), zinc(II) chloride (55 mg, 0.4 mmol) and 5 mL of aceto­nitrile was stirred for 4 h at room temperature to obtain an orange–red solid. To dissolve the precipitate, 40 µL of tri­ethyl­amine were added to the reaction vessel, forming a yellow solution. After filtering the solution and slow evaporation, transparent yellow–green crystals were obtained (yield 68%).

^1^H NMR (600 MHz, *d*_6_–DMSO, δ ppm): 9.03 (*s*, 1H, CH_imine_); 8.63 (*s*, 1H, NH_amminium salt_), 7.73 (*m*, 2H, Ar-H), 7.50 (*m*, 1H, Ar-H), 7.27 (*m*, 1H, Ar-H), 7.00 (*m*, 1H, Ar-H), 6.50 (*m*, 1H, Ar-H), 3.30 (*m*, 6H, CH_2_), 1.10 (*m*, 9H, CH_3_). ESI–MS: 653.3 (100%, *M* – Et_3_NH – Cl).

The ^1^H NMR and ESI–MS spectra of **ZnBS** are given in Figs. S7 and S8, respectively.

## 7. Refinement

Crystal data, data collection and structure refinement details are summarized in Table 4 ▸. Hydrogen atom H2 was located in a difference Fourier map for **ZnBS** and subsequently refined freely. All other H atoms were placed in idealized positions and refined in riding mode with N—H distance of 0.89 Å, C—H distances of 0.93 (aromatic), 0.97 (CH_2_) and 0.96 Å (CH_3_). Non-hydrogen atoms were refined anisotropically and hydrogen atoms with isotropic temperature factors fixed at 1.2 times *U*_eq_ of the parent atoms (1.5 for methyl groups).

## Supplementary Material

Crystal structure: contains datablock(s) ZnOQ, ZnBS. DOI: 10.1107/S2056989024010302/oo2008sup1.cif

Structure factors: contains datablock(s) ZnOQ. DOI: 10.1107/S2056989024010302/oo2008ZnOQsup2.hkl

Structure factors: contains datablock(s) ZnBS. DOI: 10.1107/S2056989024010302/oo2008ZnBSsup3.hkl

Spectroscopic data ligands and complexes. DOI: 10.1107/S2056989024010302/oo2008sup4.docx

CCDC references: 2392819, 2392818

Additional supporting information:  crystallographic information; 3D view; checkCIF report

## Figures and Tables

**Table 1:** Hydrogen-bond geometry (Å, °) for **ZnOQ**[Chem scheme1] *Cg*3 and *Cg*5 are the centroids of C4-C9 and C16-C21, respectively.

*D*—H⋯*A*	*D*—H	H⋯*A*	*D*⋯*A*	*D*—H⋯*A*
N3—H3⋯O1	0.88 (4)	1.92 (4)	2.807 (4)	178 (4)
C12—H12⋯*Cg*3^i^	0.93	2.78	3.553 (4)	141
C14—H14⋯*Cg*3^ii^	0.93	3.04	3.837 (4)	145
C24—H24*B*⋯*Cg*5^iii^	0.97	2.94	3.809 (5)	149

Symmetry codes: (i) 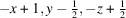; (ii) 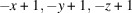; (iii) 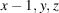.

**Table 2:** Hydrogen-bond geometry (Å, °) for **ZnBS**[Chem scheme1]

*D*—H⋯*A*	*D*—H	H⋯*A*	*D*⋯*A*	*D*—H⋯*A*
N2—H2⋯O1	0.96 (4)	1.84 (4)	2.782 (4)	165 (4)
C9—H9⋯Cl1^i^	0.93	2.83	3.752 (4)	171
C11—H11⋯Cl2^ii^	0.97	2.68	3.625 (6)	164
C13—H13⋯Cl2^iii^	0.97	2.68	3.562 (6)	151
C15—H15*A*⋯Cl1	0.97	2.80	3.684 (5)	152
C15—H15*B*⋯Cl1^iii^	0.97	2.81	3.769 (5)	169

Symmetry codes: (i) 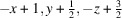; (ii) 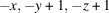; (iii) 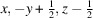.

**Table 3:** Photophysical data of the examined compounds at room temperature

Compound	Solvent (polarity)	λ_*ABS*_ (nm) / ɛ (*M*^−1^.cm^−1^.10^3^)	λ_em_ (nm)	Stokes shift (cm^−1^)	λ_em_^*b*^ (nm)/Intensity
**H2BS**	DMSO (3.96)	371 (35)	531	8122	575 / 34624
	THF (1.73)	371 (56)	534	8228	
**ZnBS**	DMSO (3.96)	372 (32)	520	7651	515 / 10616
	THF (1.73)	372 (12)	551	8878	
**HOQ**	DMSO (3.96)	264 (34); 312 (32)	528	13112	
	THF (1.73)	247 (60); 295 (56)	533	15136	
**ZnOQ**	DMSO (3.96)	265 (25); 297 (35); 450 (3)^*a*^	518	2917	
	THF (1.73)	243 (48); 307 (35); 380 (6)^*a*^	467	4902	

Notes: (*a*) shoulder excited; (*b*) in the solid state.

**Table 4:** Experimental details

	**ZnOQ**	**ZnBS**
Crystal data		
Chemical formula	(C_6_H_16_N){Zn(C_21_H_13_N_2_O_3_)Cl_2_]	(C_6_H_16_N)_2_[Zn_2_(C_20_H_14_N_2_O_2_)Cl_4_]
*M*_r_	579.80	791.24
Crystal system, space group	Monoclinic, *P*2_1_/*c*	Monoclinic, *P*2_1_/*c*
Temperature (K)	293	293
*a*, *b*, *c* (Å)	10.4571 (5), 13.9115 (5), 18.5703 (10)	11.9843 (9), 13.4570 (7), 11.9944 (11)
β (°)	100.372 (5)	103.838 (9)
*V* (Å^3^)	2657.4 (2)	1878.2 (3)
*Z*	4	2
Radiation type	Mo *K*α	Mo *K*α
μ (mm^−1^)	1.16	1.59
Crystal size (mm)	0.5 × 0.3 × 0.2	0.5 × 0.5 × 0.5

Data collection		
Diffractometer	SuperNova, Single source at offset/far, Eos	SuperNova, Single source at offset/far, Eos
Absorption correction	Multi-scan (*CrysAlis PRO*; Rigaku OD, 2018 ▸)	Multi-scan (*CrysAlis PRO*; Rigaku OD, 2018 ▸)
*T*_min_, *T*_max_	0.728, 1.000	0.473, 1.000
No. of measured, independent and observed [*I* > 2σ(*I*)] reflections	27564, 5416, 4166	10514, 3814, 2836
*R*_int_	0.042	0.044
(sin θ/λ)_max_ (Å^−1^)	0.625	0.625

Refinement		
*R*[*F*^2^ > 2σ(*F*^2^)], *wR*(*F*^2^), *S*	0.043, 0.113, 1.03	0.053, 0.158, 1.05
No. of reflections	5416	3814
No. of parameters	331	206
H-atom treatment	H atoms treated by a mixture of independent and constrained refinement	H atoms treated by a mixture of independent and constrained refinement
Δρ_max_, Δρ_min_ (e Å^−3^)	0.70, −0.38	0.93, −0.39

Computer programs: *CrysAlis PRO* (Rigaku OD, 2018 ▸), *SHELXT2014/5* (Sheldrick, 2015*a* ▸), *SHELXL2016/4* (Sheldrick, 2015*b* ▸) and *OLEX2* (Dolomanov *et al.*, 2009 ▸).

## supplementary crystallographic information

### Dichlorido[2-(4-nitrophenyl)-4-phenylquinolin-8-olato]zinc(II) (ZnOQ) Crystal data

**Table:**

(C_6_H_16_N){Zn(C_21_H_13_N_2_O_3_)Cl_2_]	*F*(000) = 1200
*M**_r_* = 579.80	*D*_x_ = 1.449 Mg m^−^^3^
Monoclinic, *P*2_1_/*c*	Mo *K*α radiation, λ = 0.71073 Å
*a* = 10.4571 (5) Å	Cell parameters from 8040 reflections
*b* = 13.9115 (5) Å	θ = 2.8–25.8°
*c* = 18.5703 (10) Å	µ = 1.16 mm^−^^1^
β = 100.372 (5)°	*T* = 293 K
*V* = 2657.4 (2) Å^3^	Block, brown
*Z* = 4	0.5 × 0.3 × 0.2 mm

### Dichlorido[2-(4-nitrophenyl)-4-phenylquinolin-8-olato]zinc(II) (ZnOQ) Data collection

**Table:**

SuperNova, Single source at offset/far, Eos diffractometer	5416 independent reflections
Radiation source: micro-focus sealed X-ray tube, SuperNova (Mo) X-ray Source	4166 reflections with *I* > 2σ(*I*)
Mirror monochromator	*R*_int_ = 0.042
Detector resolution: 15.9631 pixels mm^-1^	θ_max_ = 26.4°, θ_min_ = 2.5°
ω scans	*h* = −13→13
Absorption correction: multi-scan (CrysAlisPro; Rigaku OD, 2018)	*k* = −17→17
*T*_min_ = 0.728, *T*_max_ = 1.000	*l* = −23→23
27564 measured reflections	

### Dichlorido[2-(4-nitrophenyl)-4-phenylquinolin-8-olato]zinc(II) (ZnOQ) Refinement

**Table:**

Refinement on *F*^2^	Primary atom site location: dual
Least-squares matrix: full	Hydrogen site location: mixed
*R*[*F*^2^ > 2σ(*F*^2^)] = 0.043	H atoms treated by a mixture of independent and constrained refinement
*wR*(*F*^2^) = 0.113	*w* = 1/[σ^2^(*F*_o_^2^) + (0.045*P*)^2^ + 2.5402*P*] where *P* = (*F*_o_^2^ + 2*F*_c_^2^)/3
*S* = 1.03	(Δ/σ)_max_ = 0.001
5416 reflections	Δρ_max_ = 0.70 e Å^−^^3^
331 parameters	Δρ_min_ = −0.38 e Å^−^^3^
0 restraints	

### Dichlorido[2-(4-nitrophenyl)-4-phenylquinolin-8-olato]zinc(II) (ZnOQ) Special details

**Table:**

Geometry. All esds (except the esd in the dihedral angle between two l.s. planes) are estimated using the full covariance matrix. The cell esds are taken into account individually in the estimation of esds in distances, angles and torsion angles; correlations between esds in cell parameters are only used when they are defined by crystal symmetry. An approximate (isotropic) treatment of cell esds is used for estimating esds involving l.s. planes.

### Dichlorido[2-(4-nitrophenyl)-4-phenylquinolin-8-olato]zinc(II) (ZnOQ) Fractional atomic coordinates and isotropic or equivalent isotropic displacement parameters (Å^2^)

**Table:**

	*x*	*y*	*z*	*U*_iso_*/*U*_eq_	
Zn1	0.28872 (3)	0.61247 (2)	0.31683 (2)	0.03891 (12)	
Cl1	0.17475 (9)	0.51997 (7)	0.37894 (5)	0.0576 (2)	
O1	0.2901 (2)	0.75402 (14)	0.33465 (14)	0.0497 (6)	
N1	0.4792 (2)	0.62069 (15)	0.37204 (12)	0.0310 (5)	
C1	0.5666 (3)	0.55364 (19)	0.39637 (15)	0.0309 (6)	
Cl2	0.25071 (10)	0.58325 (7)	0.19686 (5)	0.0625 (3)	
O2	0.3841 (3)	0.14048 (19)	0.26870 (18)	0.0795 (9)	
N2	0.4125 (3)	0.16534 (19)	0.3326 (2)	0.0556 (8)	
C2	0.6879 (3)	0.5769 (2)	0.43761 (16)	0.0353 (6)	
H2	0.744674	0.527740	0.456108	0.042*	
O3	0.4033 (3)	0.11346 (17)	0.38411 (19)	0.0855 (10)	
C3	0.7251 (3)	0.6709 (2)	0.45149 (15)	0.0338 (6)	
C4	0.6353 (3)	0.7449 (2)	0.42257 (16)	0.0346 (6)	
C5	0.6627 (3)	0.8446 (2)	0.42810 (19)	0.0442 (8)	
H5	0.745372	0.865861	0.449013	0.053*	
C6	0.5672 (3)	0.9091 (2)	0.4026 (2)	0.0500 (8)	
H6	0.586516	0.974437	0.405572	0.060*	
C7	0.4418 (3)	0.8802 (2)	0.3722 (2)	0.0483 (8)	
H7	0.378430	0.926606	0.357652	0.058*	
C8	0.4088 (3)	0.7843 (2)	0.36310 (18)	0.0402 (7)	
C9	0.5106 (3)	0.71540 (19)	0.38649 (15)	0.0328 (6)	
C10	0.5301 (3)	0.45145 (19)	0.37934 (15)	0.0320 (6)	
C11	0.4760 (3)	0.4245 (2)	0.30856 (17)	0.0386 (7)	
H11	0.463670	0.470366	0.271535	0.046*	
C12	0.4404 (3)	0.3306 (2)	0.29243 (18)	0.0440 (7)	
H12	0.405316	0.312273	0.244813	0.053*	
C13	0.4580 (3)	0.26455 (19)	0.34855 (18)	0.0405 (7)	
C14	0.5133 (3)	0.2881 (2)	0.41919 (18)	0.0433 (7)	
H14	0.524837	0.241872	0.455936	0.052*	
C15	0.5514 (3)	0.3825 (2)	0.43439 (17)	0.0381 (7)	
H15	0.591252	0.399781	0.481491	0.046*	
C16	0.8549 (3)	0.6934 (2)	0.49497 (16)	0.0373 (7)	
C17	0.9647 (3)	0.6483 (2)	0.47914 (18)	0.0426 (7)	
H17	0.956210	0.602554	0.442039	0.051*	
C18	1.0869 (3)	0.6706 (2)	0.5180 (2)	0.0540 (9)	
H18	1.160042	0.640609	0.506368	0.065*	
C19	1.1004 (3)	0.7367 (3)	0.5737 (2)	0.0567 (9)	
H19	1.182556	0.752005	0.599441	0.068*	
C20	0.9924 (3)	0.7802 (3)	0.59125 (19)	0.0544 (9)	
H20	1.001479	0.824286	0.629504	0.065*	
C21	0.8703 (3)	0.7590 (2)	0.55239 (17)	0.0462 (8)	
H21	0.797671	0.788795	0.564764	0.055*	
N3	0.0767 (3)	0.87944 (19)	0.31940 (17)	0.0474 (7)	
H3	0.145 (4)	0.841 (3)	0.3240 (19)	0.057*	
C22	0.1087 (4)	0.9707 (3)	0.2842 (2)	0.0616 (10)	
H22A	0.170320	1.007137	0.319001	0.074*	
H22B	0.030357	1.008919	0.271428	0.074*	
C23	0.1659 (4)	0.9534 (3)	0.2159 (2)	0.0664 (10)	
H23A	0.098712	0.931949	0.176996	0.100*	
H23B	0.232443	0.905184	0.225731	0.100*	
H23C	0.202762	1.012092	0.201695	0.100*	
C24	0.0430 (5)	0.8986 (3)	0.3938 (3)	0.0767 (12)	
H24A	−0.025594	0.946422	0.388551	0.092*	
H24B	0.009135	0.839939	0.411421	0.092*	
C25	0.1544 (5)	0.9329 (4)	0.4497 (2)	0.0923 (15)	
H25A	0.225333	0.888423	0.452799	0.139*	
H25B	0.127844	0.937375	0.496448	0.139*	
H25C	0.181659	0.995074	0.435801	0.139*	
C26	−0.0375 (5)	0.8324 (4)	0.2696 (3)	0.1022 (17)	
H26A	−0.114192	0.871618	0.269622	0.123*	
H26B	−0.019911	0.831970	0.220101	0.123*	
C27	−0.0658 (5)	0.7353 (3)	0.2895 (3)	0.0954 (16)	
H27A	−0.125867	0.706441	0.250202	0.143*	
H27B	−0.103592	0.736367	0.332907	0.143*	
H27C	0.013090	0.698463	0.298443	0.143*	

### Dichlorido[2-(4-nitrophenyl)-4-phenylquinolin-8-olato]zinc(II) (ZnOQ) Atomic displacement parameters (Å^2^)

**Table:**

	*U*^11^	*U*^22^	*U*^33^	*U*^12^	*U*^13^	*U*^23^
Zn1	0.0347 (2)	0.02971 (19)	0.0491 (2)	0.00194 (14)	−0.00099 (15)	−0.00165 (15)
Cl1	0.0515 (5)	0.0583 (5)	0.0652 (6)	−0.0095 (4)	0.0166 (4)	−0.0010 (4)
O1	0.0354 (12)	0.0274 (10)	0.0807 (16)	0.0054 (9)	−0.0048 (11)	−0.0021 (11)
N1	0.0323 (12)	0.0226 (11)	0.0377 (13)	0.0017 (9)	0.0053 (10)	−0.0002 (10)
C1	0.0342 (15)	0.0242 (13)	0.0347 (14)	0.0018 (11)	0.0077 (12)	0.0005 (11)
Cl2	0.0686 (6)	0.0675 (6)	0.0451 (5)	0.0157 (5)	−0.0061 (4)	0.0039 (4)
O2	0.094 (2)	0.0442 (15)	0.096 (2)	−0.0164 (14)	0.0075 (18)	−0.0254 (15)
N2	0.0536 (18)	0.0287 (14)	0.088 (2)	0.0003 (13)	0.0215 (17)	−0.0089 (16)
C2	0.0335 (15)	0.0281 (14)	0.0429 (16)	0.0045 (12)	0.0033 (13)	−0.0006 (12)
O3	0.127 (3)	0.0299 (13)	0.109 (3)	−0.0107 (15)	0.046 (2)	0.0033 (15)
C3	0.0312 (14)	0.0335 (14)	0.0362 (15)	0.0015 (12)	0.0052 (12)	−0.0037 (12)
C4	0.0320 (15)	0.0301 (14)	0.0417 (16)	0.0006 (12)	0.0065 (12)	−0.0014 (12)
C5	0.0398 (17)	0.0305 (15)	0.061 (2)	−0.0055 (13)	0.0049 (15)	−0.0029 (14)
C6	0.053 (2)	0.0232 (14)	0.072 (2)	−0.0038 (14)	0.0077 (17)	−0.0020 (15)
C7	0.0447 (18)	0.0264 (14)	0.071 (2)	0.0056 (13)	0.0039 (16)	0.0023 (15)
C8	0.0389 (17)	0.0286 (14)	0.0517 (18)	0.0040 (13)	0.0043 (14)	0.0002 (13)
C9	0.0363 (15)	0.0248 (13)	0.0374 (15)	0.0009 (11)	0.0064 (12)	−0.0014 (12)
C10	0.0290 (14)	0.0249 (13)	0.0422 (16)	0.0037 (11)	0.0066 (12)	−0.0028 (12)
C11	0.0439 (17)	0.0282 (14)	0.0419 (16)	0.0026 (13)	0.0031 (13)	0.0021 (13)
C12	0.0461 (18)	0.0354 (16)	0.0488 (18)	0.0018 (14)	0.0034 (15)	−0.0090 (14)
C13	0.0397 (17)	0.0232 (13)	0.061 (2)	0.0013 (12)	0.0142 (15)	−0.0068 (14)
C14	0.0517 (19)	0.0275 (14)	0.0531 (19)	0.0081 (13)	0.0158 (15)	0.0088 (14)
C15	0.0407 (16)	0.0334 (15)	0.0406 (16)	0.0045 (13)	0.0084 (13)	0.0018 (13)
C16	0.0361 (16)	0.0333 (15)	0.0410 (16)	−0.0030 (12)	0.0027 (13)	0.0000 (13)
C17	0.0395 (17)	0.0353 (15)	0.0506 (18)	0.0024 (13)	0.0015 (14)	−0.0052 (14)
C18	0.0375 (18)	0.0501 (19)	0.070 (2)	0.0072 (15)	−0.0023 (16)	−0.0028 (18)
C19	0.0406 (19)	0.055 (2)	0.067 (2)	−0.0060 (16)	−0.0117 (17)	−0.0032 (18)
C20	0.056 (2)	0.054 (2)	0.049 (2)	−0.0070 (17)	−0.0011 (16)	−0.0154 (17)
C21	0.0415 (18)	0.0480 (18)	0.0480 (18)	0.0014 (14)	0.0048 (15)	−0.0114 (15)
N3	0.0412 (15)	0.0396 (15)	0.0621 (18)	−0.0017 (12)	0.0112 (14)	0.0001 (13)
C22	0.060 (2)	0.048 (2)	0.076 (3)	0.0071 (17)	0.009 (2)	0.0089 (19)
C23	0.065 (2)	0.074 (3)	0.059 (2)	−0.008 (2)	0.010 (2)	0.010 (2)
C24	0.082 (3)	0.070 (3)	0.085 (3)	0.009 (2)	0.034 (3)	0.001 (2)
C25	0.126 (4)	0.091 (3)	0.064 (3)	−0.024 (3)	0.028 (3)	−0.016 (3)
C26	0.097 (4)	0.087 (4)	0.118 (4)	−0.032 (3)	0.007 (3)	−0.008 (3)
C27	0.104 (4)	0.079 (3)	0.100 (4)	−0.025 (3)	0.011 (3)	0.007 (3)

### Dichlorido[2-(4-nitrophenyl)-4-phenylquinolin-8-olato]zinc(II) (ZnOQ) Geometric parameters (Å, º)

**Table:**

Zn1—Cl1	2.2140 (10)	C15—H15	0.9300
Zn1—O1	1.996 (2)	C16—C17	1.386 (4)
Zn1—N1	2.073 (2)	C16—C21	1.390 (4)
Zn1—Cl2	2.2289 (10)	C17—H17	0.9300
O1—C8	1.327 (4)	C17—C18	1.385 (4)
N1—C1	1.327 (3)	C18—H18	0.9300
N1—C9	1.373 (3)	C18—C19	1.372 (5)
C1—C2	1.396 (4)	C19—H19	0.9300
C1—C10	1.491 (4)	C19—C20	1.371 (5)
O2—N2	1.220 (4)	C20—H20	0.9300
N2—O3	1.216 (4)	C20—C21	1.381 (4)
N2—C13	1.472 (4)	C21—H21	0.9300
C2—H2	0.9300	N3—H3	0.89 (4)
C2—C3	1.376 (4)	N3—C22	1.493 (4)
C3—C4	1.432 (4)	N3—C24	1.509 (5)
C3—C16	1.482 (4)	N3—C26	1.519 (5)
C4—C5	1.415 (4)	C22—H22A	0.9700
C4—C9	1.416 (4)	C22—H22B	0.9700
C5—H5	0.9300	C22—C23	1.515 (5)
C5—C6	1.363 (4)	C23—H23A	0.9600
C6—H6	0.9300	C23—H23B	0.9600
C6—C7	1.391 (5)	C23—H23C	0.9600
C7—H7	0.9300	C24—H24A	0.9700
C7—C8	1.380 (4)	C24—H24B	0.9700
C8—C9	1.440 (4)	C24—C25	1.493 (6)
C10—C11	1.386 (4)	C25—H25A	0.9600
C10—C15	1.390 (4)	C25—H25B	0.9600
C11—H11	0.9300	C25—H25C	0.9600
C11—C12	1.376 (4)	C26—H26A	0.9700
C12—H12	0.9300	C26—H26B	0.9700
C12—C13	1.377 (4)	C26—C27	1.446 (6)
C13—C14	1.375 (4)	C27—H27A	0.9600
C14—H14	0.9300	C27—H27B	0.9600
C14—C15	1.387 (4)	C27—H27C	0.9600
			
Cl1—Zn1—Cl2	113.51 (4)	C17—C16—C21	118.3 (3)
O1—Zn1—Cl1	118.40 (8)	C21—C16—C3	121.7 (3)
O1—Zn1—N1	83.44 (8)	C16—C17—H17	119.7
O1—Zn1—Cl2	109.89 (8)	C18—C17—C16	120.7 (3)
N1—Zn1—Cl1	109.50 (7)	C18—C17—H17	119.7
N1—Zn1—Cl2	119.15 (7)	C17—C18—H18	119.9
C8—O1—Zn1	110.95 (17)	C19—C18—C17	120.2 (3)
C1—N1—Zn1	132.16 (18)	C19—C18—H18	119.9
C1—N1—C9	118.9 (2)	C18—C19—H19	120.1
C9—N1—Zn1	108.92 (17)	C20—C19—C18	119.9 (3)
N1—C1—C2	121.7 (2)	C20—C19—H19	120.1
N1—C1—C10	117.6 (2)	C19—C20—H20	119.9
C2—C1—C10	120.6 (2)	C19—C20—C21	120.3 (3)
O2—N2—C13	118.4 (3)	C21—C20—H20	119.9
O3—N2—O2	123.8 (3)	C16—C21—H21	119.7
O3—N2—C13	117.8 (3)	C20—C21—C16	120.7 (3)
C1—C2—H2	119.3	C20—C21—H21	119.7
C3—C2—C1	121.4 (3)	C22—N3—H3	109 (2)
C3—C2—H2	119.3	C22—N3—C24	111.0 (3)
C2—C3—C4	118.0 (2)	C22—N3—C26	108.2 (3)
C2—C3—C16	120.3 (3)	C24—N3—H3	110 (2)
C4—C3—C16	121.8 (2)	C24—N3—C26	110.2 (3)
C5—C4—C3	124.5 (3)	C26—N3—H3	109 (2)
C5—C4—C9	118.4 (3)	N3—C22—H22A	109.1
C9—C4—C3	117.1 (2)	N3—C22—H22B	109.1
C4—C5—H5	120.2	N3—C22—C23	112.7 (3)
C6—C5—C4	119.6 (3)	H22A—C22—H22B	107.8
C6—C5—H5	120.2	C23—C22—H22A	109.1
C5—C6—H6	119.1	C23—C22—H22B	109.1
C5—C6—C7	121.9 (3)	C22—C23—H23A	109.5
C7—C6—H6	119.1	C22—C23—H23B	109.5
C6—C7—H7	119.1	C22—C23—H23C	109.5
C8—C7—C6	121.7 (3)	H23A—C23—H23B	109.5
C8—C7—H7	119.1	H23A—C23—H23C	109.5
O1—C8—C7	123.4 (3)	H23B—C23—H23C	109.5
O1—C8—C9	119.8 (2)	N3—C24—H24A	108.7
C7—C8—C9	116.8 (3)	N3—C24—H24B	108.7
N1—C9—C4	122.6 (2)	H24A—C24—H24B	107.6
N1—C9—C8	116.2 (2)	C25—C24—N3	114.3 (4)
C4—C9—C8	121.2 (2)	C25—C24—H24A	108.7
C11—C10—C1	120.3 (3)	C25—C24—H24B	108.7
C11—C10—C15	119.7 (3)	C24—C25—H25A	109.5
C15—C10—C1	120.0 (3)	C24—C25—H25B	109.5
C10—C11—H11	119.6	C24—C25—H25C	109.5
C12—C11—C10	120.8 (3)	H25A—C25—H25B	109.5
C12—C11—H11	119.6	H25A—C25—H25C	109.5
C11—C12—H12	120.8	H25B—C25—H25C	109.5
C11—C12—C13	118.3 (3)	N3—C26—H26A	108.5
C13—C12—H12	120.8	N3—C26—H26B	108.5
C12—C13—N2	118.6 (3)	H26A—C26—H26B	107.5
C14—C13—N2	118.8 (3)	C27—C26—N3	115.0 (4)
C14—C13—C12	122.6 (3)	C27—C26—H26A	108.5
C13—C14—H14	120.8	C27—C26—H26B	108.5
C13—C14—C15	118.5 (3)	C26—C27—H27A	109.5
C15—C14—H14	120.8	C26—C27—H27B	109.5
C10—C15—H15	120.0	C26—C27—H27C	109.5
C14—C15—C10	120.1 (3)	H27A—C27—H27B	109.5
C14—C15—H15	120.0	H27A—C27—H27C	109.5
C17—C16—C3	120.0 (3)	H27B—C27—H27C	109.5
			
Zn1—O1—C8—C7	171.4 (3)	C4—C3—C16—C21	48.1 (4)
Zn1—O1—C8—C9	−8.9 (4)	C4—C5—C6—C7	1.2 (5)
Zn1—N1—C1—C2	−175.0 (2)	C5—C4—C9—N1	173.4 (3)
Zn1—N1—C1—C10	3.8 (4)	C5—C4—C9—C8	−6.9 (4)
Zn1—N1—C9—C4	−178.8 (2)	C5—C6—C7—C8	−3.1 (6)
Zn1—N1—C9—C8	1.5 (3)	C6—C7—C8—O1	179.7 (3)
O1—C8—C9—N1	5.0 (4)	C6—C7—C8—C9	−0.1 (5)
O1—C8—C9—C4	−174.7 (3)	C7—C8—C9—N1	−175.2 (3)
N1—C1—C2—C3	−3.7 (4)	C7—C8—C9—C4	5.1 (5)
N1—C1—C10—C11	48.7 (4)	C9—N1—C1—C2	1.6 (4)
N1—C1—C10—C15	−131.9 (3)	C9—N1—C1—C10	−179.5 (2)
C1—N1—C9—C4	3.9 (4)	C9—C4—C5—C6	3.7 (5)
C1—N1—C9—C8	−175.9 (3)	C10—C1—C2—C3	177.5 (3)
C1—C2—C3—C4	0.3 (4)	C10—C11—C12—C13	1.0 (5)
C1—C2—C3—C16	−179.5 (3)	C11—C10—C15—C14	−2.9 (4)
C1—C10—C11—C12	−179.1 (3)	C11—C12—C13—N2	176.2 (3)
C1—C10—C15—C14	177.7 (3)	C11—C12—C13—C14	−2.1 (5)
O2—N2—C13—C12	13.8 (5)	C12—C13—C14—C15	0.7 (5)
O2—N2—C13—C14	−167.8 (3)	C13—C14—C15—C10	1.8 (5)
N2—C13—C14—C15	−177.6 (3)	C15—C10—C11—C12	1.5 (4)
C2—C1—C10—C11	−132.4 (3)	C16—C3—C4—C5	4.1 (5)
C2—C1—C10—C15	47.0 (4)	C16—C3—C4—C9	−175.5 (3)
C2—C3—C4—C5	−175.7 (3)	C16—C17—C18—C19	1.1 (5)
C2—C3—C4—C9	4.7 (4)	C17—C16—C21—C20	1.7 (5)
C2—C3—C16—C17	47.5 (4)	C17—C18—C19—C20	0.6 (6)
C2—C3—C16—C21	−132.1 (3)	C18—C19—C20—C21	−1.1 (6)
O3—N2—C13—C12	−164.9 (3)	C19—C20—C21—C16	−0.1 (5)
O3—N2—C13—C14	13.5 (5)	C21—C16—C17—C18	−2.2 (5)
C3—C4—C5—C6	−175.9 (3)	C22—N3—C24—C25	−67.7 (5)
C3—C4—C9—N1	−7.0 (4)	C22—N3—C26—C27	169.5 (4)
C3—C4—C9—C8	172.7 (3)	C24—N3—C22—C23	169.8 (3)
C3—C16—C17—C18	178.2 (3)	C24—N3—C26—C27	−68.9 (6)
C3—C16—C21—C20	−178.7 (3)	C26—N3—C22—C23	−69.1 (4)
C4—C3—C16—C17	−132.3 (3)	C26—N3—C24—C25	172.4 (4)

### Dichlorido[2-(4-nitrophenyl)-4-phenylquinolin-8-olato]zinc(II) (ZnOQ) Hydrogen-bond geometry (Å, º)

Cg3 and Cg5 are the centroids of C4-C9 and C16-C21, respectively.

**Table:**

*D*—H···*A*	*D*—H	H···*A*	*D*···*A*	*D*—H···*A*
N3—H3···O1	0.88 (4)	1.92 (4)	2.807 (4)	178 (4)
C12—H12···*Cg*3^i^	0.93	2.78	3.553 (4)	141
C14—H14···*Cg*3^ii^	0.93	3.04	3.837 (4)	145
C24—H24*B*···*Cg*5^iii^	0.97	2.94	3.809 (5)	149

Symmetry codes: (i) −*x*+1, *y*−1/2, −*z*+1/2; (ii) −*x*+1, −*y*+1, −*z*+1; (iii) *x*−1, *y*, *z*.

### Bis(triethylammonium) {2,2'-[1,4-phenylenebis(nitrilomethylidyne)]diphenolato}bis[dichloridozinc(II)] (ZnBS) Crystal data

**Table:**

(C_6_H_16_N)_2_[Zn_2_(C_20_H_14_N_2_O_2_)Cl_4_]	*F*(000) = 820
*M**_r_* = 791.24	*D*_x_ = 1.399 Mg m^−^^3^
Monoclinic, *P*2_1_/*c*	Mo *K*α radiation, λ = 0.71073 Å
*a* = 11.9843 (9) Å	Cell parameters from 3646 reflections
*b* = 13.4570 (7) Å	θ = 3.0–27.7°
*c* = 11.9944 (11) Å	µ = 1.59 mm^−^^1^
β = 103.838 (9)°	*T* = 293 K
*V* = 1878.2 (3) Å^3^	Block, orangish brown
*Z* = 2	0.5 × 0.5 × 0.5 mm

### Bis(triethylammonium) {2,2'-[1,4-phenylenebis(nitrilomethylidyne)]diphenolato}bis[dichloridozinc(II)] (ZnBS) Data collection

**Table:**

SuperNova, Single source at offset/far, Eos diffractometer	3814 independent reflections
Radiation source: micro-focus sealed X-ray tube, SuperNova (Mo) X-ray Source	2836 reflections with *I* > 2σ(*I*)
Mirror monochromator	*R*_int_ = 0.044
Detector resolution: 15.9631 pixels mm^-1^	θ_max_ = 26.4°, θ_min_ = 2.6°
ω scans	*h* = −14→14
Absorption correction: multi-scan (CrysAlisPro; Rigaku OD, 2018)	*k* = −16→15
*T*_min_ = 0.473, *T*_max_ = 1.000	*l* = −14→14
10514 measured reflections	

### Bis(triethylammonium) {2,2'-[1,4-phenylenebis(nitrilomethylidyne)]diphenolato}bis[dichloridozinc(II)] (ZnBS) Refinement

**Table:**

Refinement on *F*^2^	Primary atom site location: dual
Least-squares matrix: full	Hydrogen site location: mixed
*R*[*F*^2^ > 2σ(*F*^2^)] = 0.053	H atoms treated by a mixture of independent and constrained refinement
*wR*(*F*^2^) = 0.158	*w* = 1/[σ^2^(*F*_o_^2^) + (0.0813*P*)^2^ + 0.7323*P*] where *P* = (*F*_o_^2^ + 2*F*_c_^2^)/3
*S* = 1.05	(Δ/σ)_max_ < 0.001
3814 reflections	Δρ_max_ = 0.93 e Å^−^^3^
206 parameters	Δρ_min_ = −0.39 e Å^−^^3^
0 restraints	

### Bis(triethylammonium) {2,2'-[1,4-phenylenebis(nitrilomethylidyne)]diphenolato}bis[dichloridozinc(II)] (ZnBS) Special details

**Table:**

Geometry. All esds (except the esd in the dihedral angle between two l.s. planes) are estimated using the full covariance matrix. The cell esds are taken into account individually in the estimation of esds in distances, angles and torsion angles; correlations between esds in cell parameters are only used when they are defined by crystal symmetry. An approximate (isotropic) treatment of cell esds is used for estimating esds involving l.s. planes.

### Bis(triethylammonium) {2,2'-[1,4-phenylenebis(nitrilomethylidyne)]diphenolato}bis[dichloridozinc(II)] (ZnBS) Fractional atomic coordinates and isotropic or equivalent isotropic displacement parameters (Å^2^)

**Table:**

	*x*	*y*	*z*	*U*_iso_*/*U*_eq_	
Zn1	0.26755 (4)	0.45138 (3)	0.68426 (4)	0.0407 (2)	
Cl1	0.37732 (10)	0.32105 (8)	0.66656 (10)	0.0572 (3)	
O1	0.2337 (3)	0.5315 (2)	0.5445 (2)	0.0478 (7)	
N1	0.3640 (3)	0.5558 (2)	0.7831 (3)	0.0356 (7)	
C1	0.2399 (3)	0.6292 (3)	0.5442 (3)	0.0389 (9)	
Cl2	0.11116 (10)	0.41690 (10)	0.74634 (14)	0.0734 (4)	
C2	0.1859 (4)	0.6806 (3)	0.4441 (4)	0.0495 (10)	
H2A	0.146066	0.645011	0.380496	0.059*	
C3	0.1907 (4)	0.7823 (4)	0.4379 (4)	0.0577 (12)	
H3	0.153834	0.814314	0.370471	0.069*	
C4	0.2495 (4)	0.8379 (4)	0.5303 (4)	0.0619 (13)	
H4	0.252095	0.906859	0.525949	0.074*	
C5	0.3038 (4)	0.7891 (3)	0.6282 (4)	0.0600 (13)	
H5	0.345075	0.826185	0.689899	0.072*	
C6	0.3000 (3)	0.6862 (3)	0.6396 (3)	0.0399 (9)	
C7	0.3605 (3)	0.6466 (3)	0.7493 (3)	0.0423 (9)	
H7	0.401718	0.692082	0.801615	0.051*	
C8	0.4335 (3)	0.5297 (3)	0.8932 (3)	0.0370 (8)	
C9	0.5314 (3)	0.5822 (3)	0.9465 (3)	0.0421 (9)	
H9	0.553630	0.637402	0.910344	0.051*	
C10	0.5960 (4)	0.5531 (3)	1.0529 (4)	0.0422 (9)	
H10	0.660230	0.589988	1.088557	0.051*	
N2	0.2124 (3)	0.4098 (3)	0.3531 (3)	0.0542 (9)	
C11	0.1537 (5)	0.4581 (4)	0.2419 (5)	0.0652 (14)	
H11A	0.195358	0.518079	0.232368	0.078*	
H11B	0.076908	0.477601	0.246096	0.078*	
C12	0.1446 (6)	0.3922 (5)	0.1357 (5)	0.0908 (19)	
H12A	0.106666	0.428237	0.068169	0.136*	
H12B	0.101226	0.333556	0.142833	0.136*	
H12C	0.220244	0.373539	0.129589	0.136*	
C13	0.1557 (5)	0.3189 (4)	0.3848 (5)	0.0790 (16)	
H13A	0.152196	0.269200	0.325422	0.095*	
H13B	0.202858	0.292250	0.455665	0.095*	
C14	0.0394 (5)	0.3356 (5)	0.3998 (6)	0.095 (2)	
H14A	−0.009863	0.356026	0.328064	0.142*	
H14B	0.041376	0.386598	0.456219	0.142*	
H14C	0.010573	0.275206	0.424967	0.142*	
C15	0.3399 (5)	0.3890 (4)	0.3623 (4)	0.0692 (14)	
H15A	0.373086	0.358910	0.436289	0.083*	
H15B	0.346897	0.341748	0.303305	0.083*	
C16	0.4060 (6)	0.4798 (6)	0.3498 (6)	0.100 (2)	
H16A	0.389363	0.499031	0.270413	0.150*	
H16B	0.486673	0.466640	0.376430	0.150*	
H16C	0.384566	0.532625	0.394328	0.150*	
H2	0.217 (4)	0.461 (3)	0.410 (4)	0.054 (13)*	

### Bis(triethylammonium) {2,2'-[1,4-phenylenebis(nitrilomethylidyne)]diphenolato}bis[dichloridozinc(II)] (ZnBS) Atomic displacement parameters (Å^2^)

**Table:**

	*U*^11^	*U*^22^	*U*^33^	*U*^12^	*U*^13^	*U*^23^
Zn1	0.0438 (3)	0.0306 (3)	0.0447 (3)	−0.00034 (18)	0.0046 (2)	0.00142 (17)
Cl1	0.0699 (8)	0.0381 (6)	0.0628 (7)	0.0139 (5)	0.0142 (6)	0.0004 (5)
O1	0.0635 (19)	0.0363 (15)	0.0399 (16)	0.0023 (13)	0.0055 (13)	−0.0005 (11)
N1	0.0391 (17)	0.0307 (17)	0.0368 (17)	−0.0030 (13)	0.0089 (14)	0.0018 (12)
C1	0.040 (2)	0.037 (2)	0.040 (2)	0.0022 (16)	0.0120 (17)	0.0023 (16)
Cl2	0.0423 (6)	0.0692 (8)	0.1099 (11)	0.0044 (6)	0.0208 (6)	0.0289 (7)
C2	0.053 (3)	0.051 (3)	0.043 (2)	0.005 (2)	0.0069 (19)	0.0054 (18)
C3	0.062 (3)	0.056 (3)	0.056 (3)	0.013 (2)	0.015 (2)	0.021 (2)
C4	0.079 (3)	0.038 (2)	0.067 (3)	0.002 (2)	0.013 (3)	0.013 (2)
C5	0.073 (3)	0.036 (2)	0.068 (3)	−0.005 (2)	0.010 (3)	0.002 (2)
C6	0.042 (2)	0.035 (2)	0.045 (2)	−0.0018 (16)	0.0143 (18)	0.0015 (16)
C7	0.045 (2)	0.038 (2)	0.043 (2)	−0.0027 (17)	0.0067 (17)	−0.0054 (17)
C8	0.041 (2)	0.036 (2)	0.033 (2)	−0.0018 (16)	0.0085 (16)	−0.0004 (15)
C9	0.049 (2)	0.036 (2)	0.042 (2)	−0.0082 (17)	0.0109 (18)	0.0081 (16)
C10	0.045 (2)	0.040 (2)	0.040 (2)	−0.0126 (17)	0.0068 (17)	0.0005 (16)
N2	0.069 (3)	0.043 (2)	0.055 (2)	−0.0016 (18)	0.0244 (19)	−0.0083 (18)
C11	0.061 (3)	0.068 (3)	0.065 (3)	0.018 (2)	0.014 (2)	−0.002 (2)
C12	0.094 (4)	0.113 (5)	0.060 (4)	0.010 (4)	0.007 (3)	−0.013 (3)
C13	0.102 (4)	0.055 (3)	0.076 (4)	−0.014 (3)	0.013 (3)	−0.010 (3)
C14	0.063 (4)	0.103 (5)	0.123 (6)	−0.031 (3)	0.031 (4)	−0.008 (4)
C15	0.084 (4)	0.073 (4)	0.053 (3)	0.025 (3)	0.021 (3)	−0.006 (2)
C16	0.081 (4)	0.118 (6)	0.116 (6)	−0.003 (4)	0.050 (4)	−0.008 (4)

### Bis(triethylammonium) {2,2'-[1,4-phenylenebis(nitrilomethylidyne)]diphenolato}bis[dichloridozinc(II)] (ZnBS) Geometric parameters (Å, º)

**Table:**

Zn1—Cl1	2.2327 (11)	C10—H10	0.9300
Zn1—O1	1.952 (3)	N2—C11	1.499 (6)
Zn1—N1	2.015 (3)	N2—C13	1.492 (7)
Zn1—Cl2	2.2248 (13)	N2—C15	1.531 (6)
O1—C1	1.316 (4)	N2—H2	0.96 (5)
N1—C7	1.284 (5)	C11—H11A	0.9700
N1—C8	1.427 (5)	C11—H11B	0.9700
C1—C2	1.403 (5)	C11—C12	1.534 (8)
C1—C6	1.422 (5)	C12—H12A	0.9600
C2—H2A	0.9300	C12—H12B	0.9600
C2—C3	1.372 (6)	C12—H12C	0.9600
C3—H3	0.9300	C13—H13A	0.9700
C3—C4	1.383 (7)	C13—H13B	0.9700
C4—H4	0.9300	C13—C14	1.465 (8)
C4—C5	1.367 (6)	C14—H14A	0.9600
C5—H5	0.9300	C14—H14B	0.9600
C5—C6	1.394 (6)	C14—H14C	0.9600
C6—C7	1.444 (5)	C15—H15A	0.9700
C7—H7	0.9300	C15—H15B	0.9700
C8—C9	1.388 (5)	C15—C16	1.483 (9)
C8—C10^i^	1.376 (5)	C16—H16A	0.9600
C9—H9	0.9300	C16—H16B	0.9600
C9—C10	1.382 (5)	C16—H16C	0.9600
C10—C8^i^	1.376 (5)		
			
O1—Zn1—Cl1	111.00 (9)	C11—N2—H2	105 (3)
O1—Zn1—N1	95.27 (12)	C13—N2—C11	115.9 (4)
O1—Zn1—Cl2	112.58 (10)	C13—N2—C15	109.8 (4)
N1—Zn1—Cl1	109.60 (9)	C13—N2—H2	111 (3)
N1—Zn1—Cl2	111.06 (10)	C15—N2—H2	101 (3)
Cl2—Zn1—Cl1	115.51 (5)	N2—C11—H11A	108.7
C1—O1—Zn1	123.6 (2)	N2—C11—H11B	108.7
C7—N1—Zn1	120.5 (3)	N2—C11—C12	114.4 (4)
C7—N1—C8	119.6 (3)	H11A—C11—H11B	107.6
C8—N1—Zn1	119.9 (2)	C12—C11—H11A	108.7
O1—C1—C2	118.7 (3)	C12—C11—H11B	108.7
O1—C1—C6	123.6 (3)	C11—C12—H12A	109.5
C2—C1—C6	117.6 (4)	C11—C12—H12B	109.5
C1—C2—H2A	119.3	C11—C12—H12C	109.5
C3—C2—C1	121.4 (4)	H12A—C12—H12B	109.5
C3—C2—H2A	119.3	H12A—C12—H12C	109.5
C2—C3—H3	119.4	H12B—C12—H12C	109.5
C2—C3—C4	121.1 (4)	N2—C13—H13A	108.7
C4—C3—H3	119.4	N2—C13—H13B	108.7
C3—C4—H4	120.8	H13A—C13—H13B	107.6
C5—C4—C3	118.4 (4)	C14—C13—N2	114.2 (5)
C5—C4—H4	120.8	C14—C13—H13A	108.7
C4—C5—H5	118.6	C14—C13—H13B	108.7
C4—C5—C6	122.8 (4)	C13—C14—H14A	109.5
C6—C5—H5	118.6	C13—C14—H14B	109.5
C1—C6—C7	125.6 (3)	C13—C14—H14C	109.5
C5—C6—C1	118.7 (4)	H14A—C14—H14B	109.5
C5—C6—C7	115.8 (4)	H14A—C14—H14C	109.5
N1—C7—C6	127.6 (4)	H14B—C14—H14C	109.5
N1—C7—H7	116.2	N2—C15—H15A	109.0
C6—C7—H7	116.2	N2—C15—H15B	109.0
C9—C8—N1	122.9 (3)	H15A—C15—H15B	107.8
C10^i^—C8—N1	118.4 (3)	C16—C15—N2	112.9 (5)
C10^i^—C8—C9	118.7 (3)	C16—C15—H15A	109.0
C8—C9—H9	119.8	C16—C15—H15B	109.0
C10—C9—C8	120.4 (4)	C15—C16—H16A	109.5
C10—C9—H9	119.8	C15—C16—H16B	109.5
C8^i^—C10—C9	120.9 (4)	C15—C16—H16C	109.5
C8^i^—C10—H10	119.5	H16A—C16—H16B	109.5
C9—C10—H10	119.5	H16A—C16—H16C	109.5
C11—N2—C15	113.0 (4)	H16B—C16—H16C	109.5
			
Zn1—O1—C1—C2	−163.5 (3)	C4—C5—C6—C1	1.9 (7)
Zn1—O1—C1—C6	17.6 (5)	C4—C5—C6—C7	−179.1 (5)
Zn1—N1—C7—C6	−5.5 (6)	C5—C6—C7—N1	175.5 (4)
Zn1—N1—C8—C9	154.8 (3)	C6—C1—C2—C3	0.0 (6)
Zn1—N1—C8—C10^i^	−23.2 (5)	C7—N1—C8—C9	−27.6 (6)
O1—C1—C2—C3	−179.0 (4)	C7—N1—C8—C10^i^	154.5 (4)
O1—C1—C6—C5	178.0 (4)	C8—N1—C7—C6	176.9 (4)
O1—C1—C6—C7	−1.0 (6)	C8—C9—C10—C8^i^	1.6 (7)
N1—C8—C9—C10	−179.5 (4)	C10^i^—C8—C9—C10	−1.6 (7)
C1—C2—C3—C4	0.3 (7)	C11—N2—C13—C14	−61.3 (6)
C1—C6—C7—N1	−5.5 (7)	C11—N2—C15—C16	57.8 (6)
C2—C1—C6—C5	−1.0 (6)	C13—N2—C11—C12	−61.6 (6)
C2—C1—C6—C7	−180.0 (4)	C13—N2—C15—C16	−171.1 (5)
C2—C3—C4—C5	0.5 (7)	C15—N2—C11—C12	66.4 (6)
C3—C4—C5—C6	−1.6 (8)	C15—N2—C13—C14	169.1 (5)

Symmetry code: (i) −*x*+1, −*y*+1, −*z*+2.

### Bis(triethylammonium) {2,2'-[1,4-phenylenebis(nitrilomethylidyne)]diphenolato}bis[dichloridozinc(II)] (ZnBS) Hydrogen-bond geometry (Å, º)

**Table:**

*D*—H···*A*	*D*—H	H···*A*	*D*···*A*	*D*—H···*A*
N2—H2···O1	0.96 (4)	1.84 (4)	2.782 (4)	165 (4)
C9—H9···Cl1^ii^	0.93	2.83	3.752 (4)	171
C11—H11···Cl2^iii^	0.97	2.68	3.625 (6)	164
C13—H13···Cl2^iv^	0.97	2.68	3.562 (6)	151
C15—H15*A*···Cl1	0.97	2.80	3.684 (5)	152
C15—H15*B*···Cl1^iv^	0.97	2.81	3.769 (5)	169

Symmetry codes: (ii) −*x*+1, *y*+1/2, −*z*+3/2; (iii) −*x*, −*y*+1, −*z*+1; (iv) *x*, −*y*+1/2, *z*−1/2.
