# Supplementary material for: Crystal structures and photophysical properties of mono- and dinuclear ZnII complexes flanked by tri­ethyl­ammonium
Source: Acta Crystallogr E Crystallogr Commun. 2024 Oct 24;80(Pt 11):1210–6. doi: 10.1107/S2056989024010302 (PMC11660477; doi:10.1107/S2056989024010302)
Supplement: Supplementary file 4 [file e-80-01210-sup4.docx]

**SUPPORTING INFORMATION**

**Crystal structures and photophysical properties of mono- and dinuclear Zn(II) complexes flanked by triethylammonium**

**Hai Le Thi Hong^a,b^, Hien Nguyen^a^, Duong Trinh Hong^a^, Ninh Nguyen Hoang^a^, Khanh Nguyen Nhat^a^, and** Luc Van Meervelt^c*^.

^a^Department of Chemistry, Hanoi National University of Education, 136 Xuan Thuy, Cau Giay, Hanoi, Vietnam.

^b^Institute of Natural Science, Hanoi National University of Education, 136 Xuan Thuy, Cau Giay, Hanoi, Vietnam

^c^Department of Chemistry, KU Leuven, Biomolecular Architecture, Celestijnenlaan 200F, Leuven (Heverlee), B-3001, Belgium.

*Correspondence e-mail: luc.vanmeervelt@kuleuven.be

**TABLE OF CONTENT**

[Figure S1. ^1^H NMR spectrum of **HOQ** 2](#_Toc175687442)

[Figure S2. ^1^H NMR spectrum of **ZnOQ** 2](#_Toc175687443)

[Figure S3. ESI - MS spectrum of **ZnOQ** 3](#_Toc175687444)

[Figure S4. Absorption spectra of **HOQ** and **ZnOQ** in two solvents THF and DMSO 3](#_Toc175687445)

[Figure S5. Emission spectra of **HOQ** and **ZnOQ** in DMSO and THF solvents (λ_ex_ = 300nm). 4](#_Toc175687446)

[Figure S6. ^1^H NMR spectrum of **H_2_BS** 4](#_Toc175687447)

[Figure S7. ^1^H NMR spectrum of **ZnBS** 5](#_Toc175687448)

[Figure S8. ESI-MS spectrum of **ZnBS** 5](#_Toc175687449)

[Figure S9. Absorption spectra of **H_2_BS** and **ZnBS** in DMSO and THF solvents. 6](#_Toc175687450)

[Figure S10. Emission spectra of **H_2_BS** and **ZnBS** in DMSO and THF solvents (λ_ex_ = 365nm). 6](#_Toc175687451)

[Figure S11. Photograph of **H_2_BS**(a) and **ZnBS**(b) in solid state under UV lamp (λ_ex_ = 365 nm). 6](#_Toc175687452)

Table S1. Selected proton signals of ligands and complexes………………………………………………7


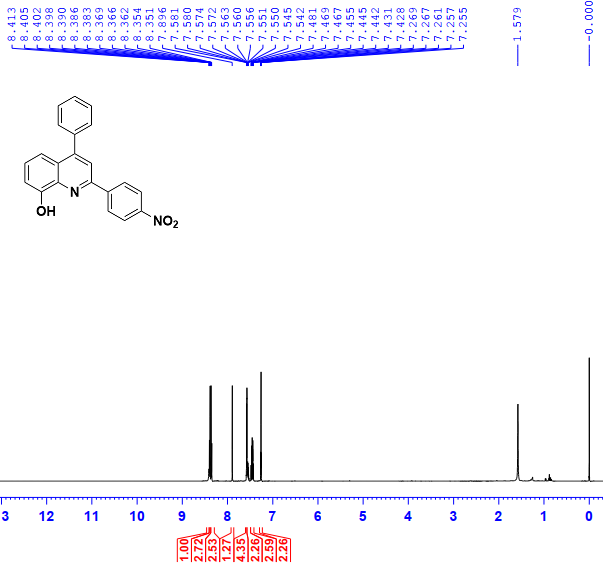


Figure S1. ^1^H NMR spectrum of **HOQ**


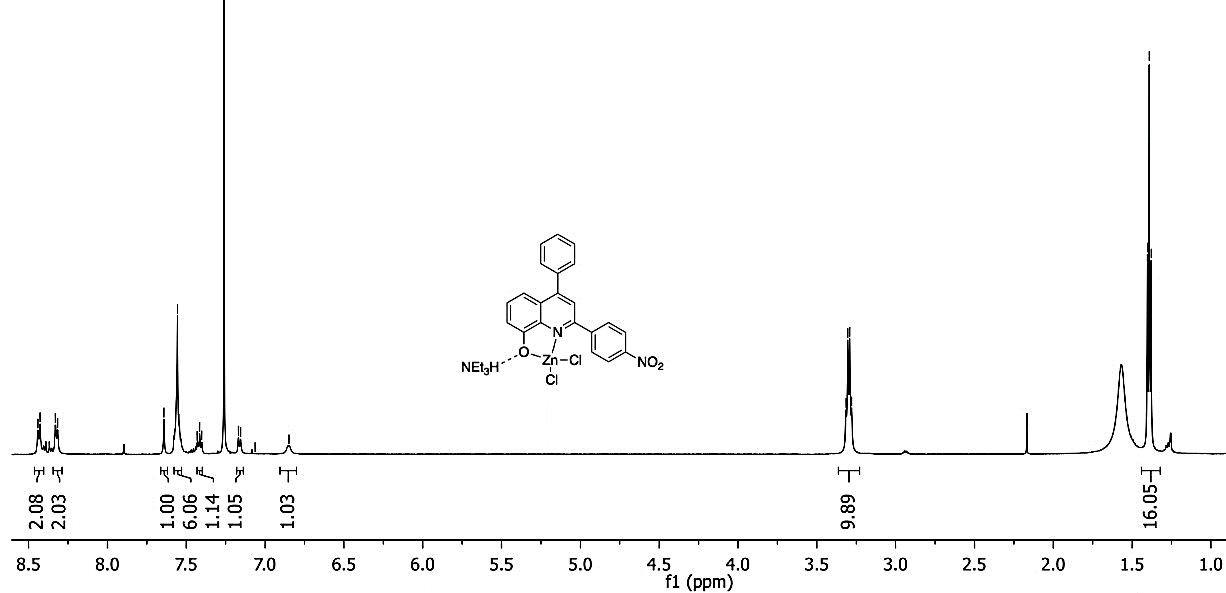


Figure S2. ^1^H NMR spectrum of **ZnOQ**


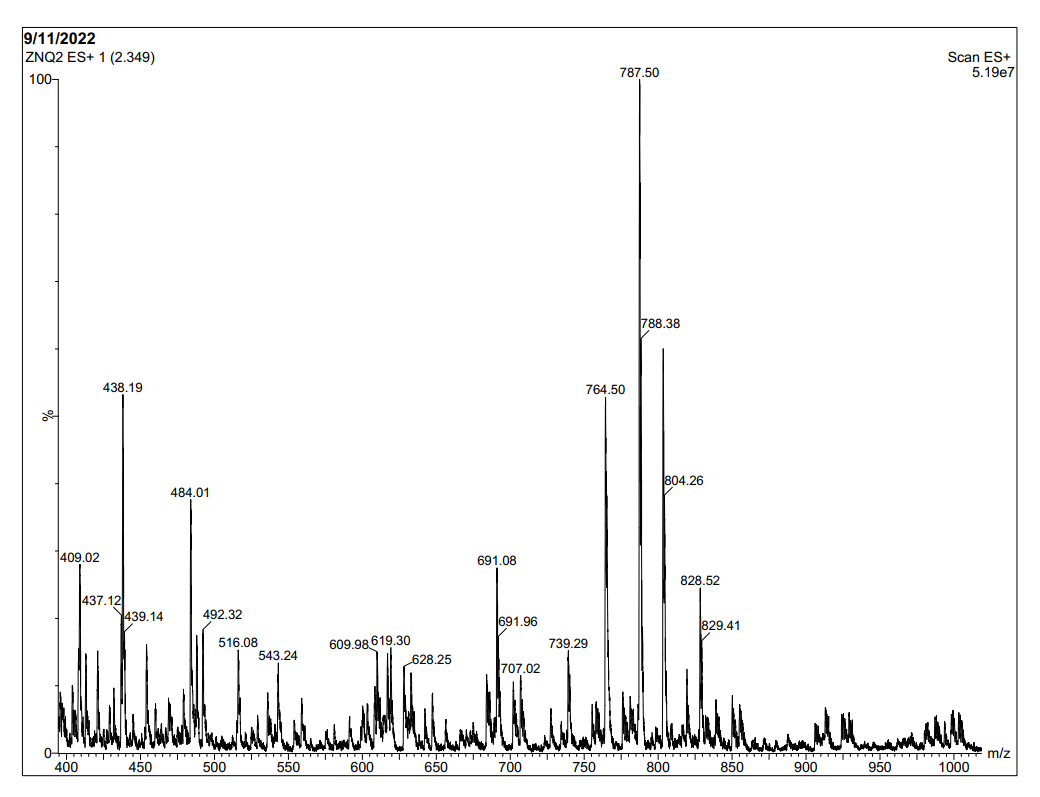


Figure S3. ESI - MS spectrum of **ZnOQ**


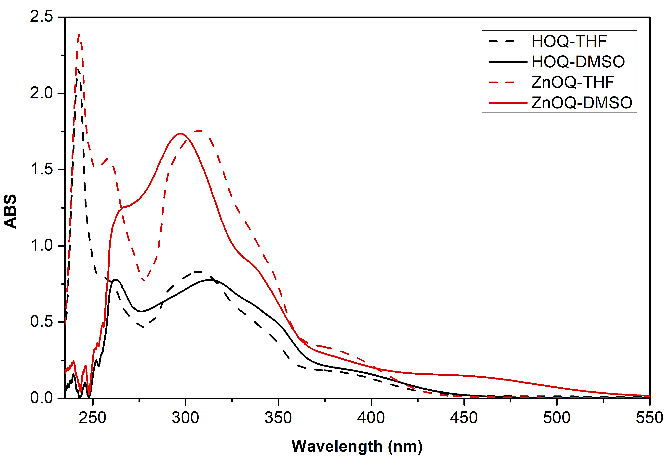


Figure S4. Absorption spectra of **HOQ** and **ZnOQ** in THF and DMSO solvents


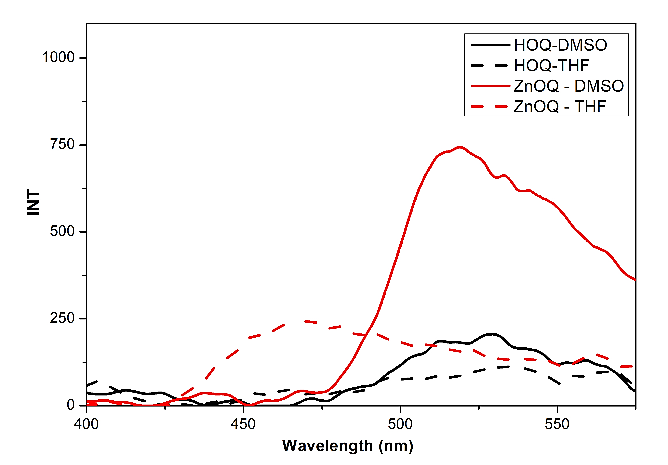


Figure S5. Emission spectra of **HOQ** and **ZnOQ** in DMSO and THF solvents (λ_ex_ = 300nm).


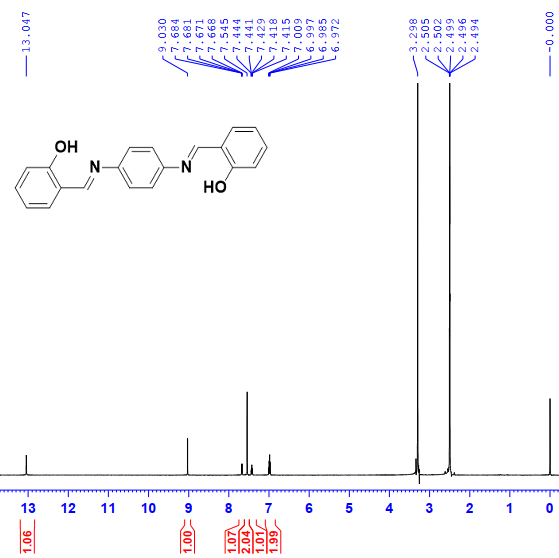


Figure S6. ^1^H NMR spectrum of **H_2_BS**


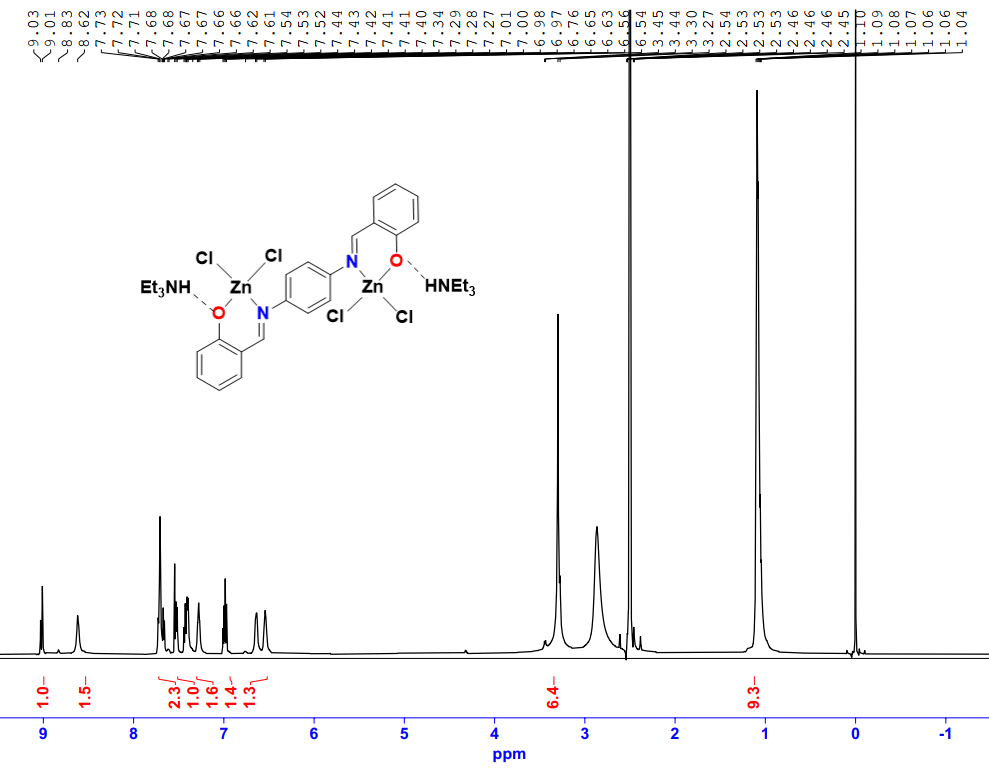


Figure S7. ^1^H NMR spectrum of **ZnBS**


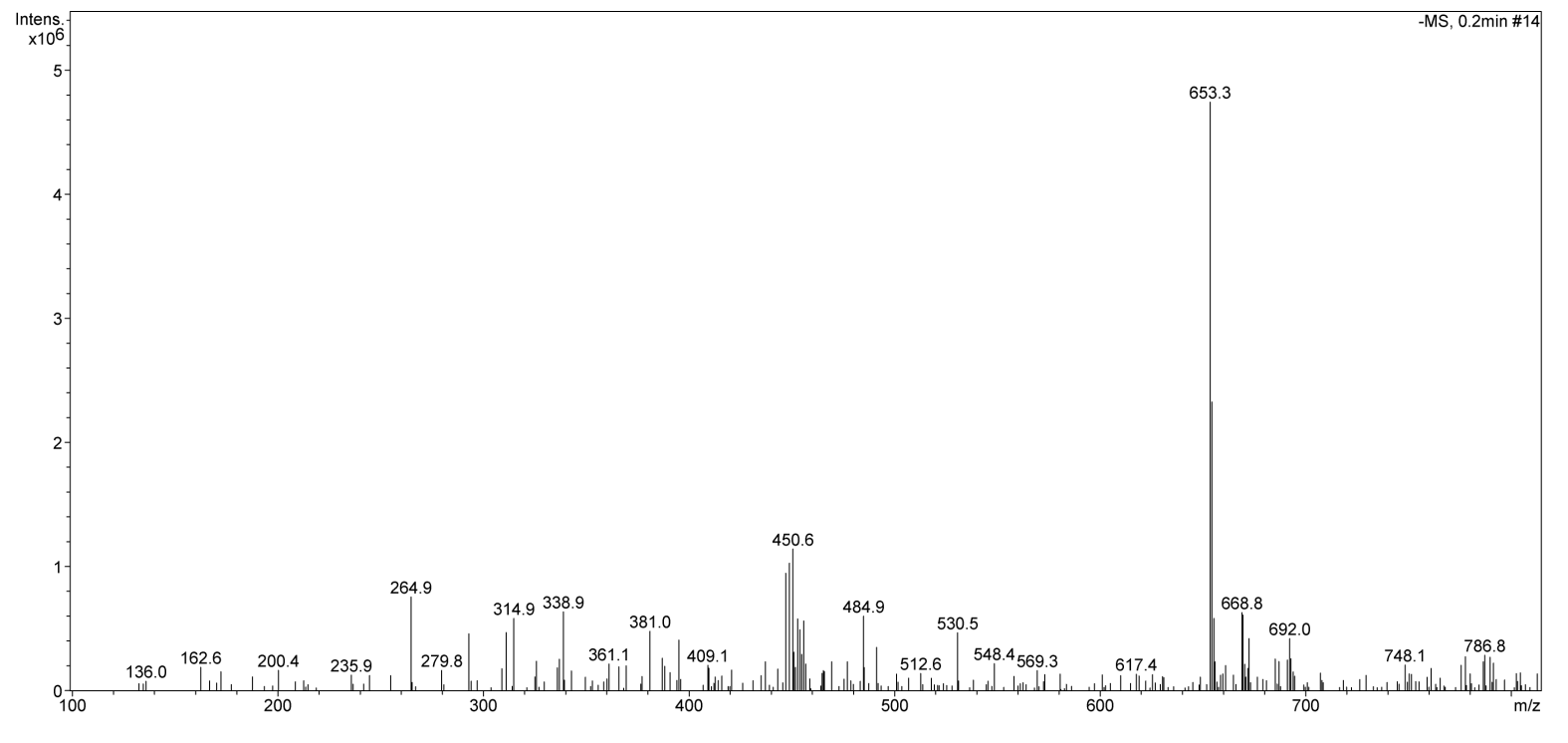


Figure S8. ESI-MS spectrum of **ZnBS**


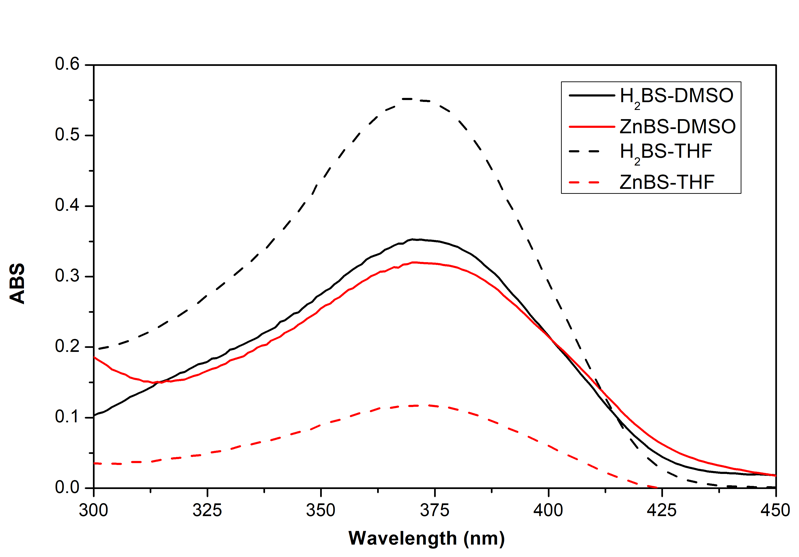


Figure S9. Absorption spectra of **H_2_BS** and **ZnBS** in DMSO and THF solvents.


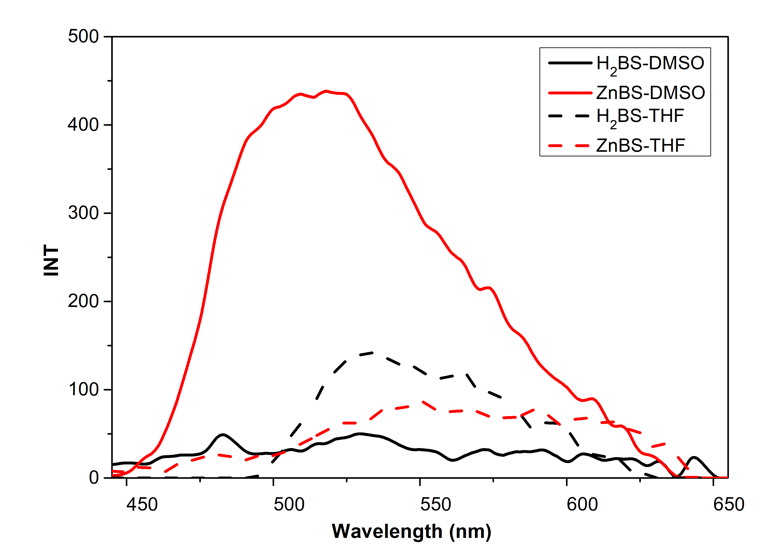


Figure S10. Emission spectra of **H_2_BS** and **ZnBS** in DMSO and THF solvents (λ_ex_ = 365nm).

| 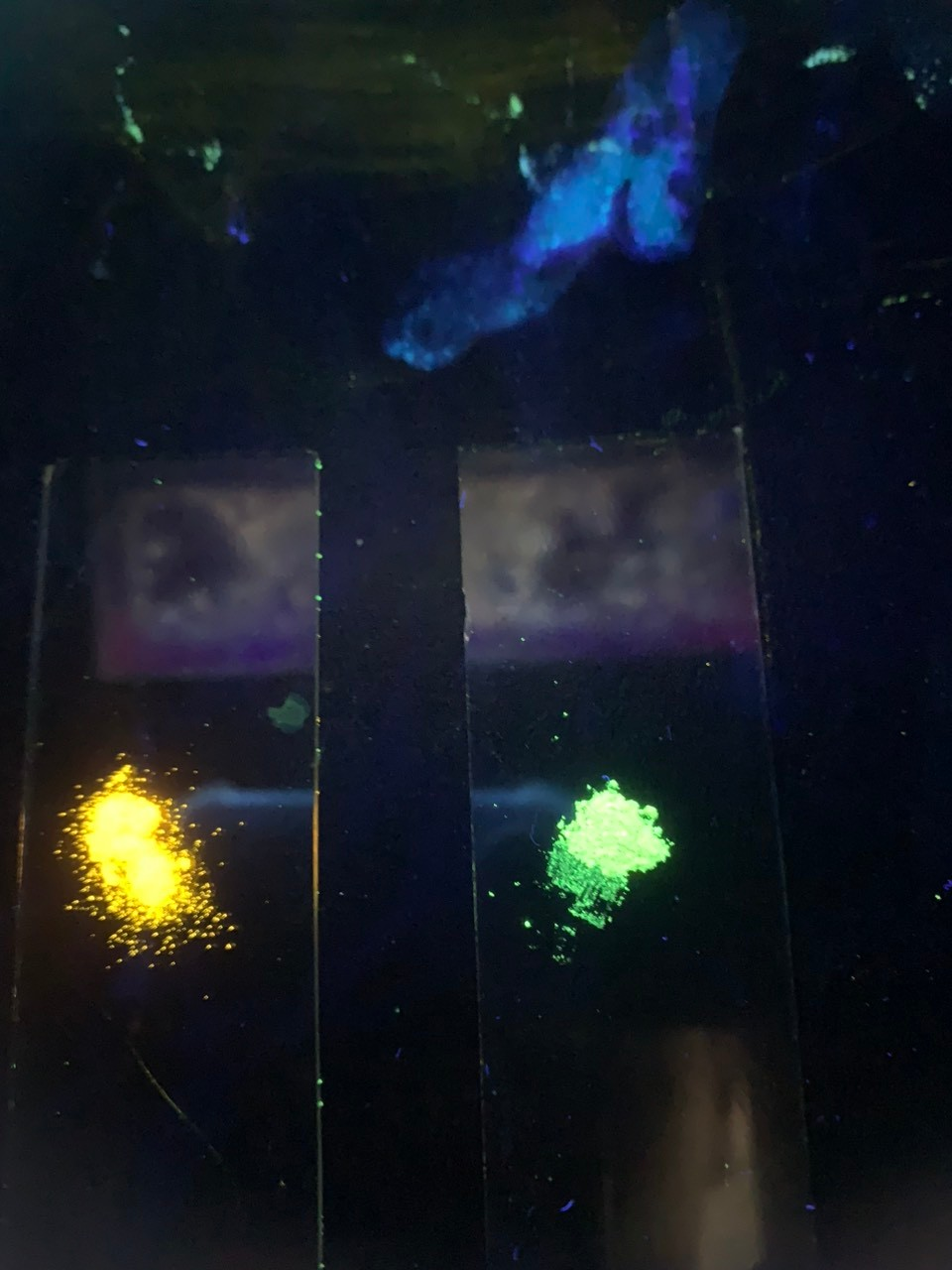 | 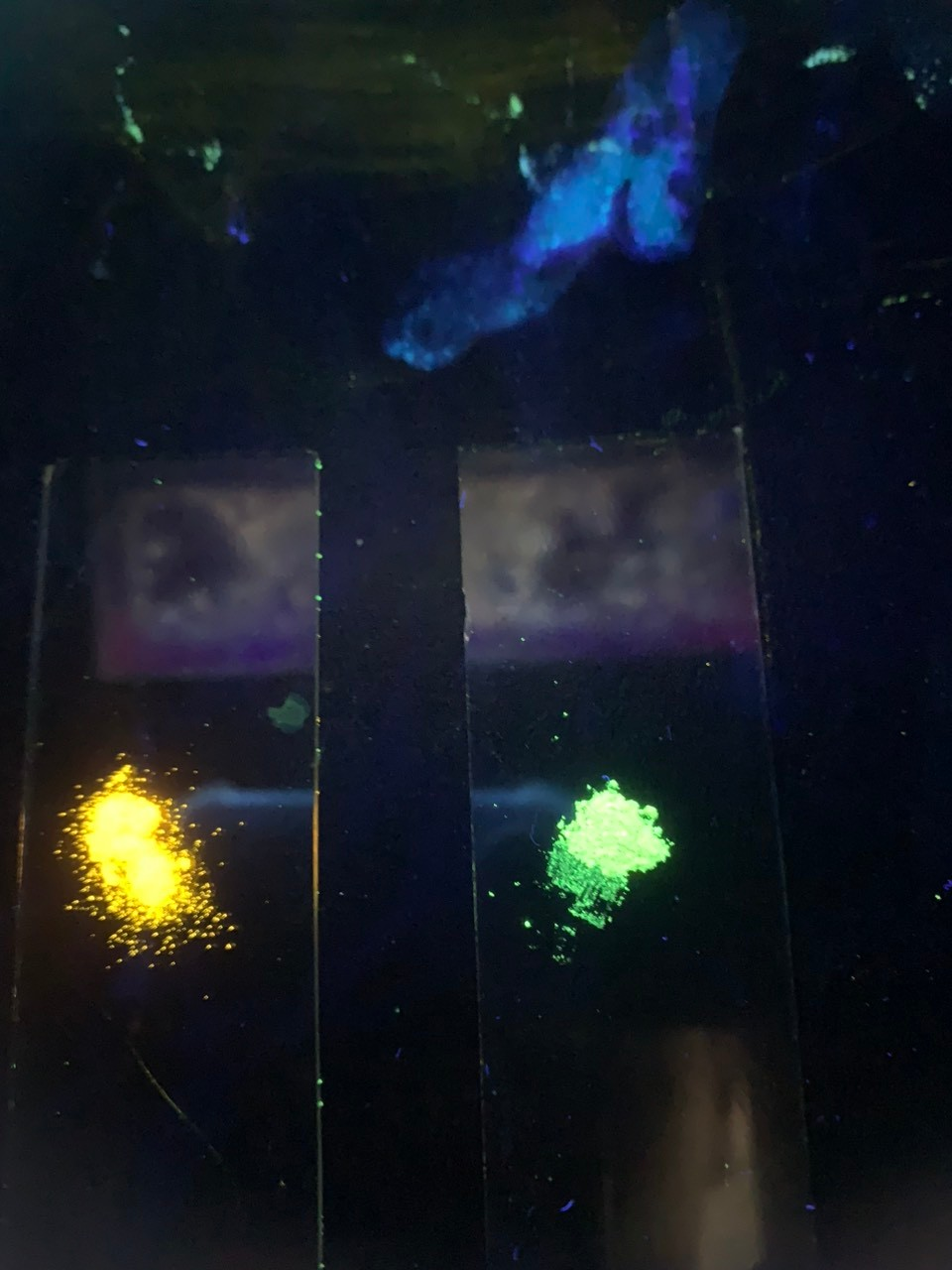 |
| --- | --- |
| a | b |

Figure S11. Photograph of **H_2_BS**(a) and **ZnBS**(b) in solid state under UV lamp (λ_ex_ = 365 nm).

Table S1. Selected proton signals of ligands and complexes (δ, ppm)

|  | **HOQ** | **ZnOQ** | **H_2_BS** | **ZnBS** |
| --- | --- | --- | --- | --- |
| H^1^ | 8.36-8.39 (m) | 8.44 (m) | 7.54 (s) | 7.73 (m) |
| H^2^ |  |  |  |  |
| H^3^ | 7.9 (s) | 7.64 (s) | 9.00 (s) | 9.03 (s) |
| NH_amminium_ | - | 6.85 (br) | - | 8.63 (s) |
| CH_3,amminium_ | - | 1.40 (t) | - | 1.10 (m) |
| CH_2,amminium_ | - | 3.31 (q) | - | 3.30 (m) |
| OH | 8.41 (br) | - | 13.00 (s) | - |

The two complexes, **ZnOQ** and **ZnBS**, were fully characterized using ESI mass spectrometry and ^1^H NMR spectroscopy. Notably, the ^1^H NMR spectra of these complexes lack the proton signals from hydroxyl groups, indicating that the OH groups are deprotonated and coordinated to Zn(II) through oxygen atoms. Additionally, the ^1^H NMR spectra of both complexes show signals corresponding to the protons of the counterion HNEt_3_^+^ (see Table S1). Furthermore, the chemical shifts of all protons near the coordination center were altered compared to those of the free ligands.
